# AMPK Is Involved in the Regulation of Incretin Receptors Expression in Pancreatic Islets under a Low Glucose Concentration

**DOI:** 10.1371/journal.pone.0064633

**Published:** 2013-05-22

**Authors:** Kazuki Tajima, Jun Shirakawa, Yu Togashi, Hideaki Inoue, Koichiro Sato, Kazuki Orime, Yuzuru Ito, Mitsuyo Kaji, Eri Sakamoto, Akinobu Nakamura, Kazutaka Aoki, Yoshio Goshima, Tatsuya Atsumi, Yasuo Terauchi

**Affiliations:** 1 Department of Endocrinology and Metabolism, Yokohama-City University, Yokohama, Japan; 2 Department of Medicine II, Hokkaido University, Sapporo, Japan; 3 Department of Molecular Pharmacology and Neurobiology, Yokohama-City University, Yokohama, Japan; University of Ulster, United Kingdom

## Abstract

The precise role of AMP-activated protein kinase (AMPK), a target of metformin, in pancreatic β cells remains controversial, even though metformin was recently shown to enhance the expression of incretin receptors (GLP-1 and GIP receptors) in pancreatic β cells. In this study, we investigated the effect of AMPK in the regulation of incretin receptors expression in pancreatic islets. The phosphorylation of AMPK in the mouse islets was decreased by increasing glucose concentrations. We showed the expression of incretin receptors in bell-shaped response to glucose; Expression of the incretin receptors in the isolated islets showed higher levels under a medium glucose concentration (11.1 mM) than that under a low glucose concentration (2.8 mM), but was suppressed under a high glucose concentration (22.2 mM). Both treatment with an AMPK inhibitor and DN-AMPK expression produced a significant increase of the incretin receptors expression under a low glucose concentration. By contrast, in hyperglycemic *db/db* islets, the enhancing effect of the AMPK inhibitor on the expression of incretin receptors was diminished under a low glucose concentration. Taken together, AMPK is involved in the regulation of incretin receptors expression in pancreatic islets under a low glucose concentration.

## Introduction

Type 2 diabetes is characterized by impaired β cell function and peripheral insulin resistance [1], [2]. In regard to β cell function, the incretin effect, i.e., the increased insulin response to glucose, is markedly reduced in patients with type 2 diabetes [3]. While the incretin effect accounts for more than 50% of the meal-related insulin secretion in healthy individuals [4], its contribution to the overall insulin response after oral glucose ingestion may amount to <20% in patients with type 2 diabetes [3]. The two main incretins are glucagon-like peptide 1 (GLP-1) [5] and glucose-dependent insulinotropic peptide (GIP) [6].

The mechanisms underlying the downregulation of the incretin effect in patients with type 2 diabetes are still incompletely understood. Impaired insulinotropic activity of the incretins may be responsible for the downregulation of the incretin receptors expression. The idea of a reduced expression of GIP receptors in patients with type 2 diabetes has already expounded in 1997 [7]. The results of previous animal studies lend support to this contention [8]–[10]. Moreover, in a recent study, exposure of β cells to high blood glucose concentrations led to downregulation of the GLP-1 receptor expression and impaired insulin secretion in response to GLP-1, both *in vivo* and *in vitro* [8]. These studies provided the rationale for the design of new therapies, based on increment of the incretin receptor expressions.

Recently, GLP-1 receptor agonists [11] and dipeptidyl-peptidase-4 inhibitors [11] have come to be widely used as treatment targeting β cell dysfunction in patients with type 2 diabetes [12]. In addition, metformin has been increasingly used in combination with incretin-based compounds [13]. According to previous reports, metformin not only increases the plasma GLP-1 levels in rodents [14]–[16], and humans [17]–[19], but also enhances the expressions of GLP-1 and GIP receptor in INS-1 β cells and mouse islets [14], [20]. However, the molecular target of metformin is still elusive. The main effect of metformin is to decrease hepatic glucose production and metformin has been shown to inhibit gluconeogenesis through the AMPK-dependent and –independent mechanism [21]. The effects of metformin on the expression of the incretin receptors in β cells are reported to be mediated by a PPAR-α-dependent, AMPK-independent mechanism [14]. On the contrary, Pan et al. reported that 5-aminoimidazole-4-carboxamide-1-β-d-riboruranosid (AICAR) mimicked the actions of metformin on the expression of the incretin receptors [20]. The interactions between metformin and the incretin axis also remain to be clearly elucidated.

AMPK is a heterotrimeric serine/threonine protein kinase that acts as a metabolic sensor of the cellular energy status [22]. AMPK activity is activated by a wide array of metabolic stresses, including hypoxia, ischemia, and oxidative stresses [23]. In general, activation of AMPK triggers glucose uptake and lipid oxidation to produce energy, while turning off energy-consuming processes including glucose and lipid production to restore energy balance. AMPK activity is regulated both allosterically by AMP, and through reversible phosphorylation at Thr-172 of the α-subunit by an upstream kinase. AMPK controls whole-body glucose homeostasis by regulating metabolism in multiple peripheral tissues, such as skeletal muscle, liver, adipose tissue, and pancreatic β cells-key tissues in pathogenesis of type 2 diabetes. While AMPK activation in the muscle and liver improves the insulin sensitivity and is now established as a strategy for glucose lowering [23], the role of AMPK in the pancreatic β cells is controversial [24]. It has been reported that elevated glucose concentrations suppress AMPK phosphorylation and activation, and that the AMPK activity is inversely correlated with insulin release [25]–[28]. However, other studies have shown that insulin release is normal in isolated islets from mice lacking either the α1 or α2 catalytic subunit of AMPK [29]–[31]. Despite its profound effect on insulin release, the role of AMPK in β cell is unclear and downstream targets of AMPK that mediate these physiological processes remain to be identified. Thus, it also remains to be established whether AMPK plays a role in the regulation of incretin receptors expression. In the present study, we examined the effects of AMPK on the incretin receptors expression in mouse pancreatic islets.

## Materials and Methods

### Ethics statement

This study was approved by the Animal Care Committee of the Yokohama City University (Permit Number: F11-28, F11-29). All the animal procedures were performed in accordance with the institutional animal care guidelines and the guidelines of the Animal Care Committee of the Yokohama City University. Special care was taken to reduce animal suffering and to use the minimum number of animals for all studies.

### Animals

C57BL/6J male mice aged 8–9 weeks were purchased from CLEA Japan (Tokyo, Japan). Littermate db/db and db/+ mice aged 7 weeks were purchased from Charles River Japan (Yokohama, Japan). All mice were fed standard chow (MF, Oriental Yeast, Tokyo, Japan) and were allowed free access to water and the diets. The animal housing rooms were maintained at a constant room temperature (25°C) under a 12-h light (7:00 A.M.)/dark (7:00 P.M.) cycle.

### Treatment of the islets with chemicals

The islets were isolated with collagenase (Sigma-Aldrich), as described elsewhere [32]. The AMPK activator, 5-aminoimidazole-4-carboxamide-1-β-d-riboruranosid (AICAR), and the AMPK inhibitor, compound C, were purchased from Sigma-Aldrich (St. Louis, MO). The glucokinase activator (GKA), compound A, was purchased from Calbiochem (Darmstadt, Germany). Isolated islets were incubated for 24 hours in RPMI1640 medium containing 2.8 or 11.1 mM glucose and 30 µM compound A, 1 mM AICAR, 40 µM compound C, or vehicle.

### Glucose-stimulated insulin secretion by islets

After culture for 24 hours in RPMI 1640 medium at 2.8 mM glucose supplemented with 10% fetal bovine serum and vehicle or 40 µM compound C, 10 islets were incubated at 37°C for 1 h in Krebs-Ringer bicarbonate (KRB) buffer containing 2.8 mM glucose. The buffer was then replaced with fresh KRB buffer containing 2.8 mM glucose with or without 10 nM GLP-1 and 10 nM GIP (Sigma-Aldrich) for 1.5 h, followed by replacement with KRB buffer containing 11.1 mM glucose with or without 10 nM GLP-1 and 10 nM GIP for an additional 1.5 h. Compound C (40 µM) was added to the KRB/glucose for throughout the study.

### Western blot analysis

The isolated islets (100 islets per mouse) were incubated in each well of 12-well plate. After culture for 24 hours, the isolated islets (100 islets per sample) were lysed, as described elsewhere [32]. After centrifugation, the extracts were subjected to immunoblotting with antibodies to AMPKα, p-AMPKα (Cell signaling Technology), anti-GIPR antibody (Santa Cruz Biotechnology, CA, USA), anti-GLP-1R antibody, GAPDH (Abcam, MA, USA). Densitometry was performed using the Multi gauge V3.0 software (Fuji Film Life Science, Tokyo, Japan).

### Real-time PCR

Total RNA extraction, cDNA synthesis, and real-time quantitative PCR were performed as described previously [33]. cDNA was prepared using the High Capacity cDNA Reverse Transcription Kits (Applied Biosystems, CA, USA) and quantitative PCR was performed by 7900 real-time PCR system (Applied Biosystems). All the probes were purchased from Applied Biosystems. The data were normalized to the mRNA expression level of β actin and to the control samples.

### Suppression of AMPK phosphorylation by adenovirus-mediated expression of a mutant form of AMPK

AdCMV-LacZ and Ad-AMPK-α2-K45R, a dominant-inhibitory AMPK, were kindly provided by Dr. Birnbaum (Howard Hughes Medical Institute and Department of Medicine, University of Pennsylvania school of Medicine, Philadelphia, Pennsylvania, USA). Trypsinized islet cells were cultured in monolayer, and infected with AdCMV-LacZ or Ad-AMPK-α2-K45R viruses at 250 m.o.i. for 24 h in 1.5 ml of RPMI 1640 medium containing 2.8 mM glucose.

### Statistical analyses

Results are expressed as means ± S.E. (*n*). Differences between two groups were analyzed for statistical significance by Student's *t* test. Individual comparisons among more than 2 groups were assessed with the post-hoc Fisher's PLSD test. *P*<0.05 was considered statistically significant.

## Results

### The effect of glucose signal on the phosphorylation level of AMPK and the expression of the incretin receptors in mouse islets

**Figure 1:**
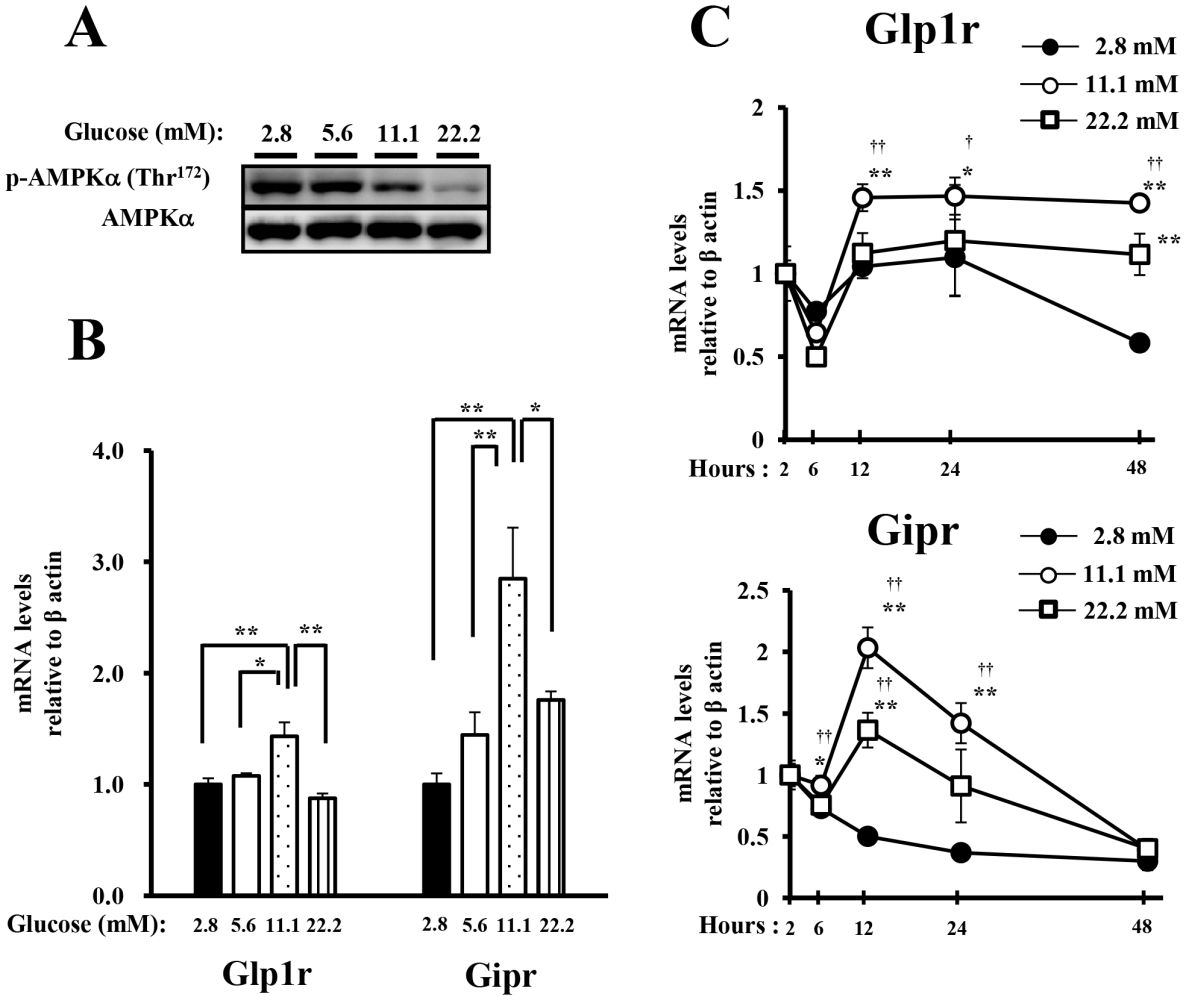
The effect of glucose signal on the phosphorylation level of AMPK and the expressions of the incretin receptors in mouse islets. A), B) Isolated islets were incubated for 24 h in the presence of 2.8, 5.6, 11.1 or 22.2 mM glucose. A) Total cell extracts from the isolated islets were subjected to immunoblotting for p-AMPKα (Thr^172^) and AMPKα. Data shown are representative of three independent experiments. B) The GLP-1 and GIP receptor expressions in the islets were determined by real-time quantitative RT-PCR and normalized to the expression level of β actin mRNA and to the samples at 2.8 mM glucose (n = 4). The samples are from independent experiments. *P<0.05 and ** P<0.01. C) Isolated islets were incubated for the indicated times in the presence of 2.8, 11.1 or 22.2 mM glucose. The GLP-1 and GIP receptor expressions were determined as shown in B. (n = 4). The samples are from independent experiments. * P<0.05 and ** P<0.01 vs. 2.8 mM glucose. ^†^ P<0.05 and ^††^ P<0.01 vs. 22.2 mM glucose.

We first confirmed that the phosphorylation of endogenous AMPKα on Thr172 in the mouse islets was decreased by increasing glucose concentrations (Figure 1A). We next evaluated whether the glucose signal regulated the expression of the incretin receptors (GLP-1 and GIP receptors). Expression of the incretin receptors at 11.1 mM glucose showed higher levels than that at 2.8 or 5.6 mM glucose, but was suppressed under high glucose concentration (22.2 mM) (Figure 1B). Moreover, when the islets were incubated at 2.8, 11.1 or 22.2 mM glucose for up to 48 h, the expression of the incretin receptors at 11.1 mM glucose was higher than those at 2.8 mM glucose, however, the expression was suppressed at 22.2 mM glucose at any of the indicated times (Figure 1C).

### The effect of glucokinase activator on the expression of the incretin receptors under a low glucose concentration

**Figure 2:**
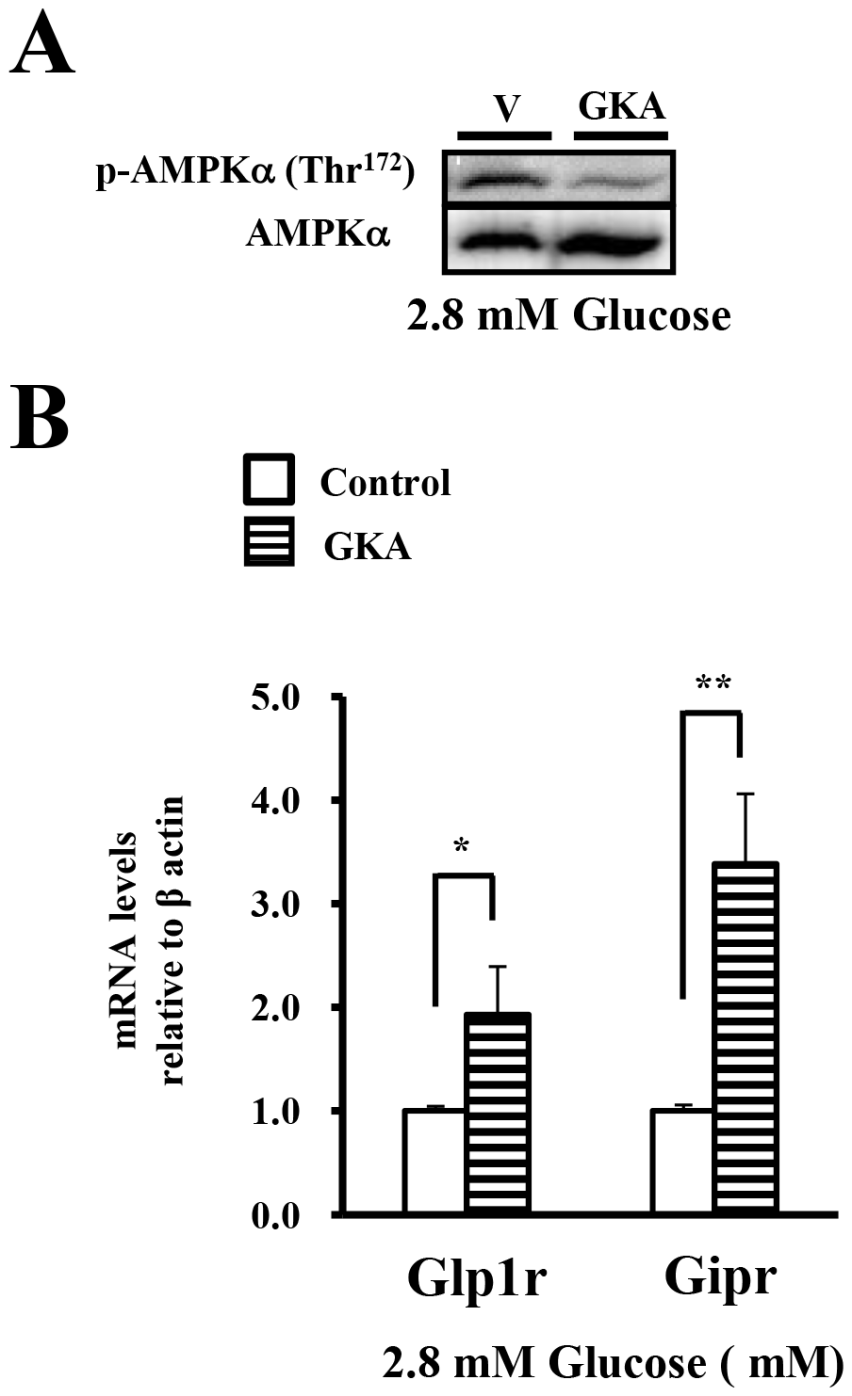
The effect of glucokinase activator on the expression of the incretin receptors under a low glucose concentration. A), B). Isolated islets were incubated with DMSO (ctl) or GKA (G: 30 µM compound A) in the presence of 2.8 mM glucose for 24 hours. Total cell extracts from the isolated islets were subjected to immunoblotting for p-AMPKα (Thr^172^) and AMPKα. Data shown are representative of three independent experiments. B) The GLP-1 and GIP receptor expressions were determined by real-time quantitative RT-PCR and normalized to the β actin mRNA level and to the control samples (n = 4). *P<0.05, ** P<0.01.

It has shown that glucokinase, the main enzyme involved in the phosphorylation of glucose in the β cells [34], plays a crucial role in the glucose-induced dephosphorylation of AMPK [35]. Glucokinase activator (GKA) is an allosteric activator of glucokinase in the β cells and liver [36]. To assess whether the glucose signal might be involved in the expression of the incretin receptors, we treated isolated islets with GKA under low glucose concentration (2.8 mM). Treatment with GKA produced dephosphorylation of AMPK in the islets at 2.8 mM glucose (Figure 2A). The expression of the incretin receptors was significantly increased by treatment with GKA at 2.8 mM glucose (Figure 2B).

### The effect of pharmacologic modulation of AMPK phosphorylation on the expression of the incretin receptors under a low glucose concentration

**Figure 3:**
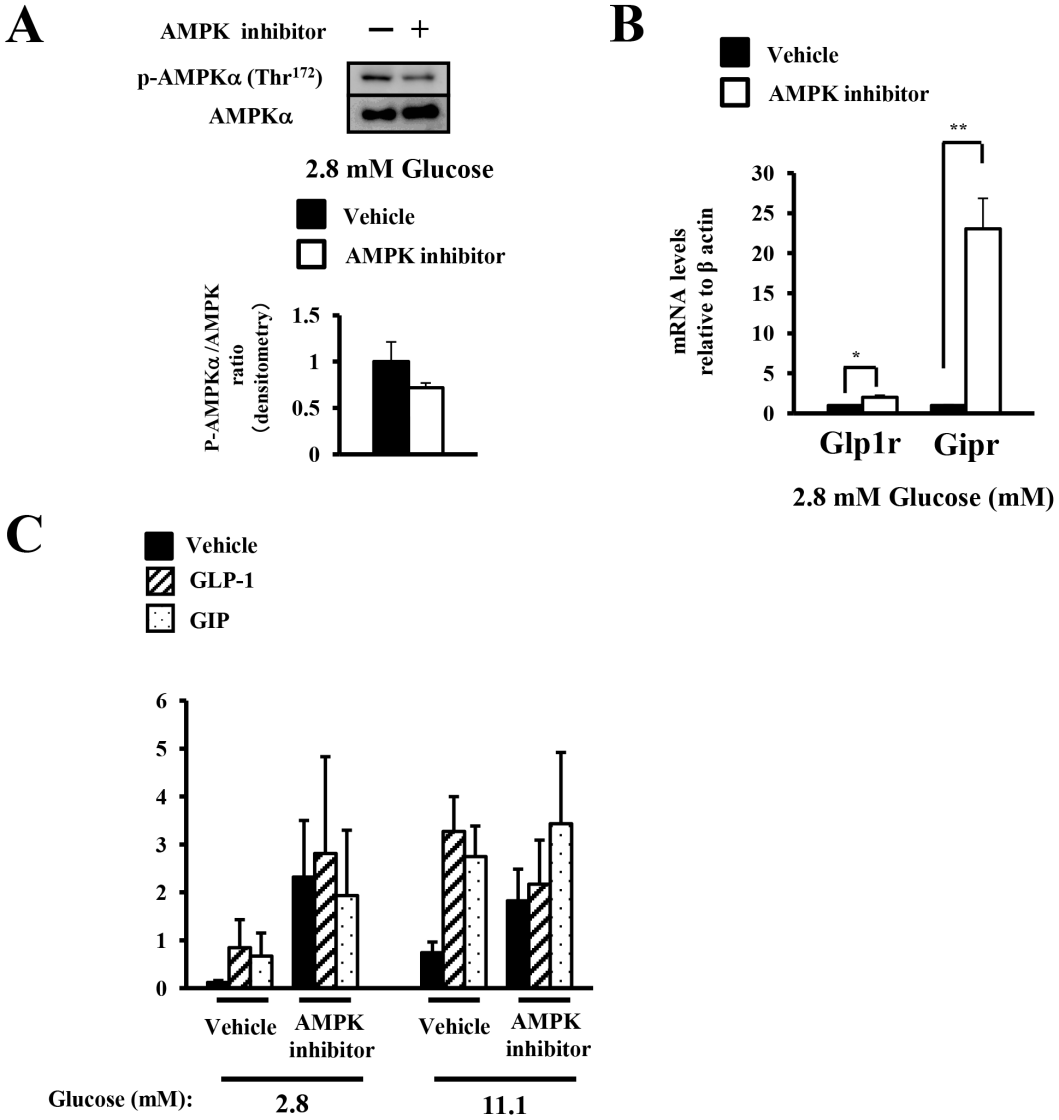
The effect of pharmacologic modulation of AMPK phosphorylation on the expressions of the incretin receptors. A)–C) Isolated islets were treated for 24 h with vehicle (Veh; DMSO) or the AMPK inhibitor (AMPKi; 40 µM compound C) in the presence of 2.8 mM glucose. A) Total islet extracts from the isolated islets were subjected to immunoblotting for p-AMPKα (Thr^172^) and AMPKα (n = 3). Data shown are representative of three independent experiments. B) The GLP-1 and GIP receptor expressions were determined by real-time quantitative RT-PCR and normalized to the expression level of β actin mRNA and normalized to the vehicle-treated samples (n = 4–7). C) Insulin secretion by the islets treated with the AMPK inhibitor or vehicle (ctl) in the presence of 2.8 mM glucose for 24 h with or without addition of 10 nM GLP-1 or 10 nM GIP. The results are expressed in ng of insulin/10 islets/90 min (n = 3). *P<0.05 and ** P<0.01.

To assess whether the phosphorylation state of AMPK regulated the expression of the incretin receptors in the islets under lower glucose concentrations (2.8–11.1 mM), we treated isolated islets with an AMPK inhibitor under a low glucose concentration (2.8 mM) to activate the endogenous AMPK. The AMPK inhibitor decreased the phosphorylation level of AMPK to 30% (Figure 3A). Treatment with the AMPK inhibitor resulted in a significant increase in the expression of the incretin receptors as compared with the levels in the control at 2.8 mM glucose (Figure 3B). These results suggested that the expression of the incretin receptors was dependent, at least in part, on the phosphorylation state of AMPK under low glucose concentrations.

To investigate the effect of AMPK inhibitor on the sensitivity of the islets to GLP-1 and GIP-stimulated insulin secretion, we examined insulin response to incretins at 2.8 and 11.1 mM glucose in isolated islets treated with vehicle or AMPK inhibitor at 2.8 mM glucose for 24 h. AMPK inhibitor increased insulin secretion from islet at 2.8 and 11.1 mM glucose, although the difference was not statistically significant (Figure 3C). The relative effects of GLP-1 and GIP on insulin secretion were not significantly increased by AMPK inhibitor treatment (Figure 3C).

### The impact of manipulation of AMPK phosphorylation by dominant-negative AMPK expression on the expression of the incretin receptors under a low glucose concentration

**Figure 4:**
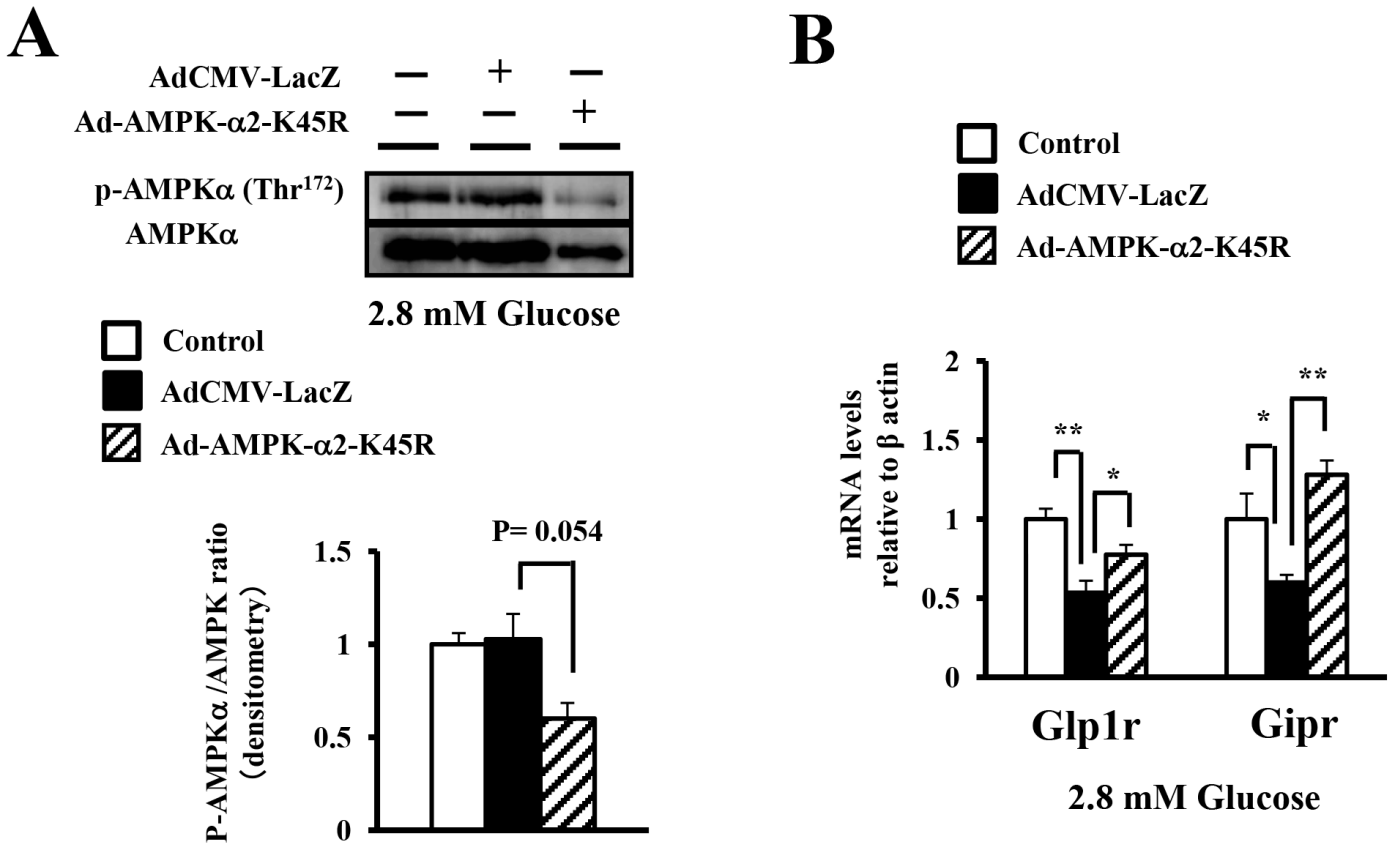
The impact of manipulation of AMPK phosphorylation by DN-AMPK expression on the expressions of the incretin receptors. A), B) Isolated islet cells in monolayer were infected with the Ad-AMPK-α2-K45R or AdCMV-LacZ virus in the presence of 2.8 mM glucose for 24 h. A) Total islet extracts from the uninfected cells and virus-infected cells were subjected to immunoblotting for p-AMPKα (Thr^172^) and AMPKα. Data shown are representative of three independent experiments. B) The GLP-1 and GIP receptor expressions were determined by real-time quantitative RT-PCR and normalized to the β actin mRNA level and to the control samples (n = 3). The samples are from independent experiments. *P<0.05 and ** P<0.01.

To confirm whether the phosphorylation state of AMPK regulated the expression of the incretin receptors in the islets, we manipulated AMPK phosphorylation in the islet cells by inducing the expression of a dominant-negative form of the AMPKα subunit (DN-AMPK) carrying a point mutation (K45R). Overexpression of DN-AMPK with adenoviral vector in isolated islets decreased the ratio of phosphorylated to total AMPK to 60% under a low glucose concentration (2.8 mM) to activate the endogenous AMPK (Figure 4A). The expression of the incretin receptors was significantly decreased in isolated islets treated with overexpression of AdCMV-LacZ compared with in control islets (Figure 4B), which was due to the direct effect of adenoviral infection. We confirmed that the morphology of trypsinized islet cells was unaffected by adenoviral infection (Figure. S1). In addition, we next evaluated the apoptosis in islets infected with the viruses using TUNEL staining. The results showed that the ratio of TUNEL-positive β cells were equally around 10% across the three groups (Mock control islet cells, islet cells infected with AdCMV-LacZ, and islet cells infected with Ad-AMPK-α2-K45R). These results indicated that the apoptosis was not induced by adenoviral infection. (Figure. S1). AMPK dephosphorylation by DN-AMPK expression led to significant increases in the expression of the incretin receptors as compared with islets treated with overexpression of AdCMV-LacZ at 2.8 mM glucose (Figure 4B). These results lend support to our hypothesis that the expression of the incretin receptors was dependent, at least in part, on the phosphorylation state of AMPK under a low glucose concentration.

### The effect of glucokinase activator on the expression of the incretin receptors under a medium glucose concentration

**Figure 5:**
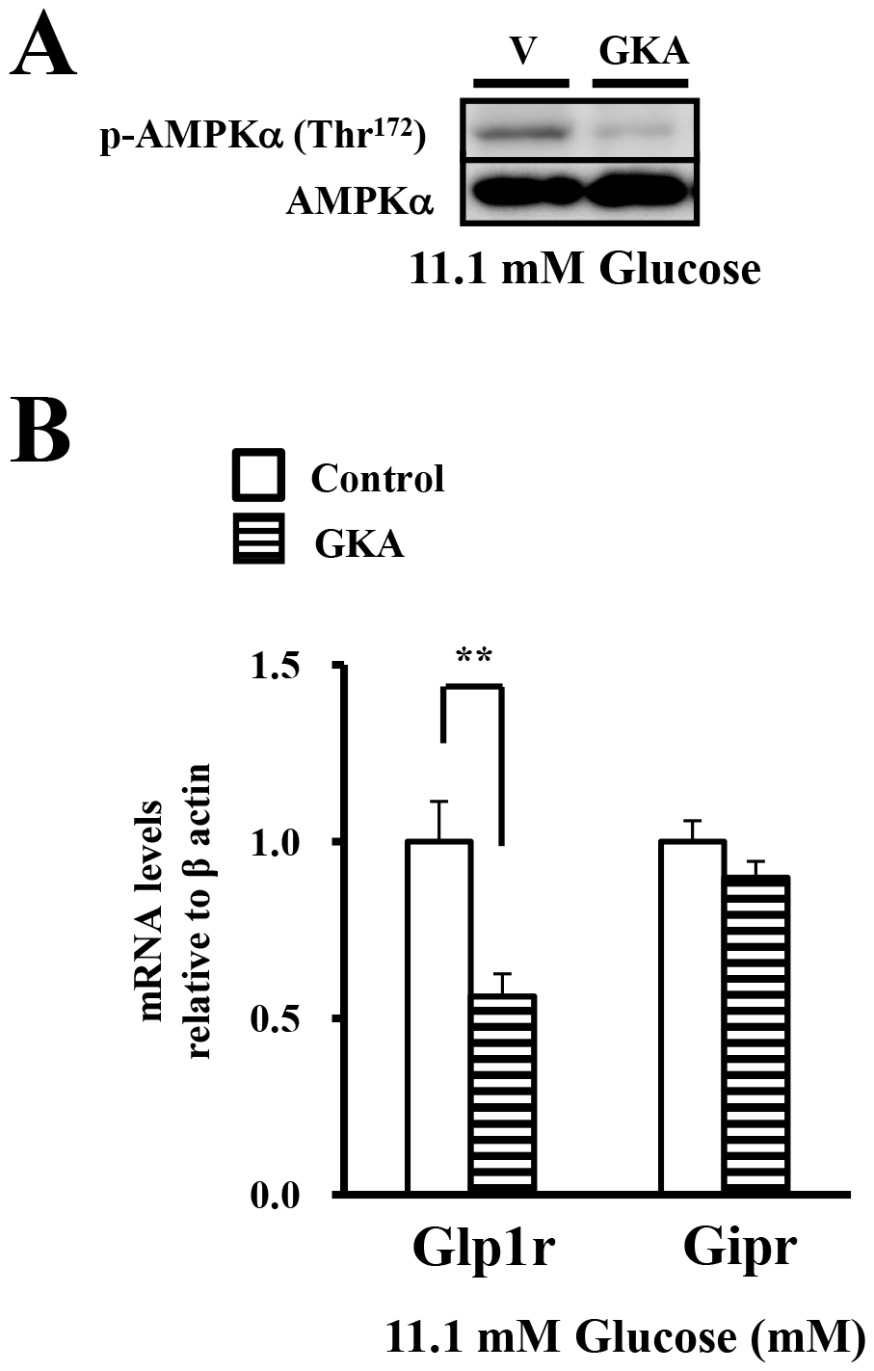
The effect of glucokinase activator on the expression of the incretin receptors under a medium glucose concentration. A), B). Isolated islets were incubated with DMSO (ctl) or GKA (G: 30 µM compound A) in the presence of 11.1 mM glucose for 24 hours. Total cell extracts from the isolated islets were subjected to immunoblotting for p-AMPKα (Thr^172^) and AMPKα. Data shown are representative of three independent experiments. B) The GLP-1 and GIP receptor expressions were determined by real-time quantitative RT-PCR and normalized to the β actin mRNA level and to the control samples (n = 4). *P<0.05, ** P<0.01.

To assess whether the glucose signal might be involved in the expression of the incretin receptors, we treated isolated islets with GKA under a medium glucose concentration (11.1 mM). Treatment with GKA produced dephosphorylation of AMPK in the islets at 11.1 mM glucose (Figure 5A). The GLP-1 receptor expression was decreased by treatment with GKA at 11.1 mM glucose, whereas no significant decrease of the GIP receptor was observed (Figure 5B). The result suggested that an excessively glucose signal suppressed the expression of GLP-1 receptor.

### The effect of pharmacologic modulation of AMPK phosphorylation on the expression of the incretin receptors under a medium glucose concentration

**Figure 6:**
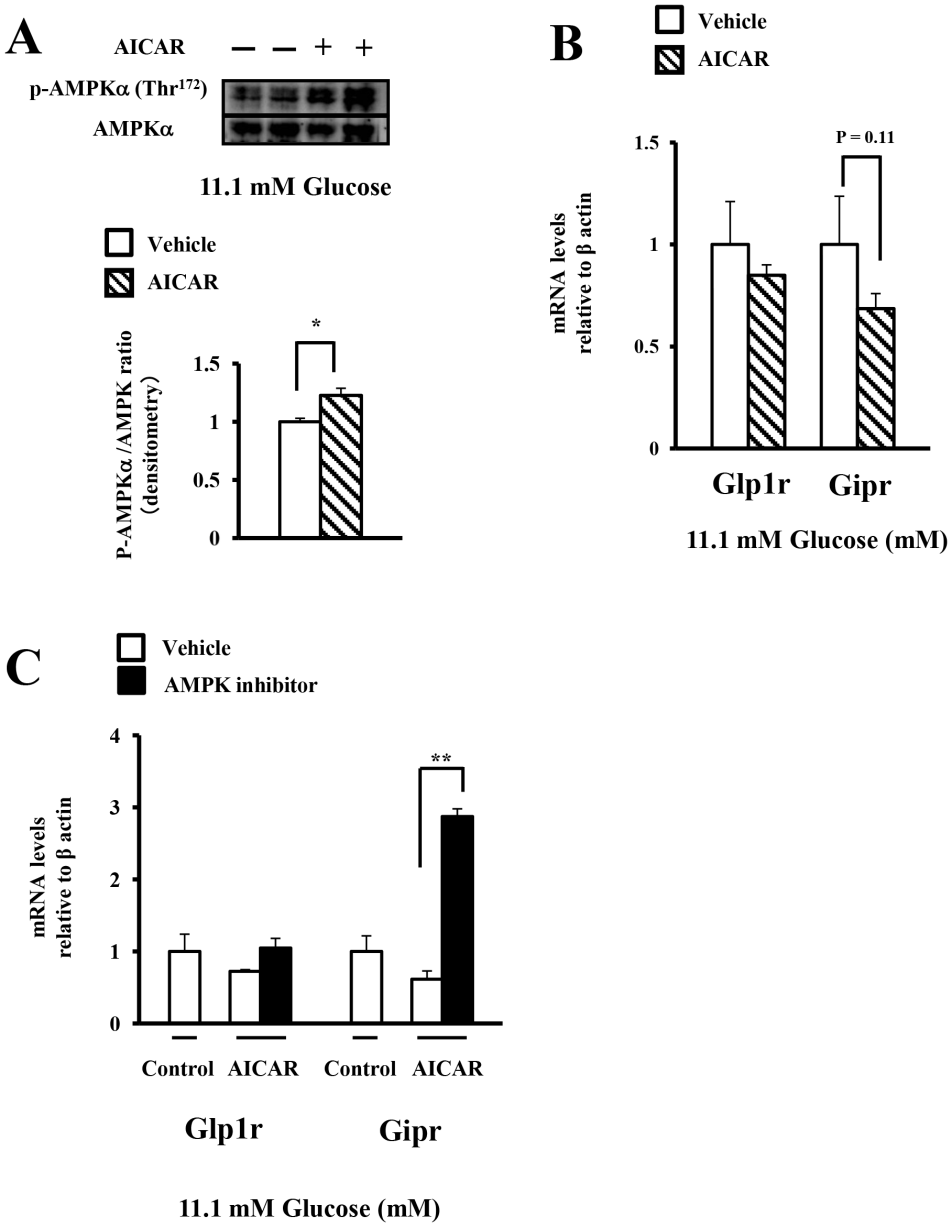
The effect of pharmacologic modulation of AMPK phosphorylation on the expression of the incretin receptors under a medium glucose concentration. A), B) Isolated islets were treated for 24 h with vehicle (V, water) or AICAR (A, 1 mM) in the presence of 11.1 mM glucose. A) Total islet extracts from the isolated islets were subjected to immunoblotting for p-AMPKα (Thr^172^) and AMPKα.(n = 4) B) The GLP-1 and GIP receptor expressions were determined by real-time quantitative RT-PCR and normalized to the β actin mRNA level and to the control samples (n = 4). C) Isolated islets were pre-treated for 15 min with vehicle (Veh; DMSO) or the AMPK inhibitor (AMPKi; 40 µM compound C) in the presence of 11.1 mM glucose, followed by the addition of vehicle (V; water) or AICAR (A, 1 mM) for an additional 24 h. The GLP-1 and GIP receptor expressions were determined as shown in B) (n = 3). White bars, vehicle; Black bars; AMPKi. *P<0.05 and ** P<0.01.

To assess whether the phosphorylation state of AMPK regulated the expression of the incretin receptors in the islets under lower glucose concentrations (2.8–11.1 mM), we treated isolated islets with AICAR under a medium glucose concentration (11.1 mM) to suppress the endogenous enzyme to some extent. AICAR increased the phosphorylation levels of AMPK at 11.1 mM glucose, as compared to that in the control (Figure 6A). The expression of the incretin receptors in the islets was not significantly decreased by treatment with AICAR at 11.1 mM glucose (Figure 6B). We next assess the expression of incretin receptors in islets co-treated with AICAR and the AMPK inhibitor. Co-treatment with the AMPK inhibitor significantly increased the expression of GIP receptor in islets treated with AICAR, but not GLP-1 receptor (Figure 6C).

### The phosphorylation levels of AMPK and the expression of the incretin receptors in *db/db* mice

**Figure 7:**
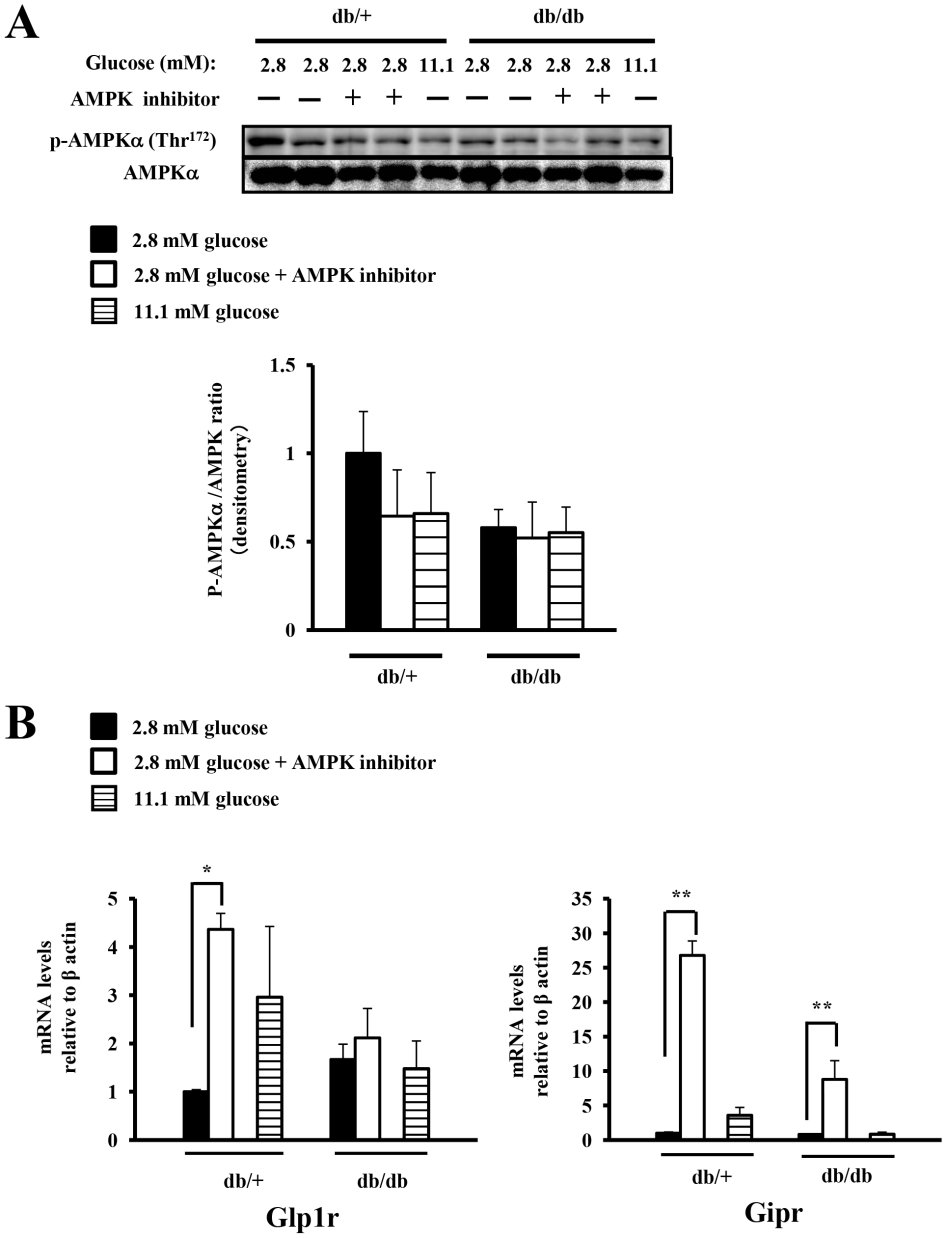
The impact of pharmacologic modulation of AMPK phosphorylation on the expressions of the incretin receptors in ***db/db*** **mice.** A), B) Isolated islets from 7-week-old *db/+* and *db/db* mice were pre-treated for 15 min with vehicle (Veh; DMSO) or an AMPK inhibitor (AMPKi; 40 µM compound C) in the presence of 2.8 mM glucose, or with vehicle (Veh; DMSO) in the presence of 11.1 mM glucose, followed by the addition of vehicle (Veh; water) for an additional 24 h. A) Total islet extracts from the isolated islets were subjected to immunoblotting for p-AMPKα (Thr^172^) and AMPKα (n = 2–4). B) The GLP-1 and GIP receptor expressions were determined by real-time quantitative RT-PCR and normalized to the β actin mRNA expression level and to the vehicle-treated control samples at 2.8 mM glucose (n = 2–3). *P<0.05 and ** P<0.01.

We next evaluated the phosphorylation levels of AMPK and the expression of the incretin receptors in the islets of the *db/db* mice, a model of chronic hyperglycemia. In the *db/db* islets, the ratio of phosphorylated-AMPK/total-AMPK at 2.8 mM glucose was decreased as compared to that in the euglycemic *db/+* islets (Figure 7A). In the *db/db* islets, expression of the incretin receptors was decreased at 11.1 mM glucose as compared with that in the euglycemic *db/+* islets, although the difference was not statistically significant (Figure 7B). In addition, we also evaluated the effect of the AMPK inhibitor on the expression of incretin receptors in the *db/db* islets under a low glucose concentration (2.8 mM) to activate the endogenous AMPK. The AMPK inhibitor decreased the ratio of phosphorylated-AMPK/total-AMPK to 64% in the *db/+* islets and to 89% in the *db/db* islets (Figure 7A). The enhancing effect of the AMPK inhibitor on the GIP receptor expression in the *db/db* islets was reduced to one third compared to the effect in the *db/+* islets. No significant increase of the GLP-1 receptor was observed in the *db/db* islets following AMPK inhibitor treatment at 2.8 mM glucose (Figure 7B). Taken together, the enhancing effect of the AMPK inhibitor on the expression of incretin receptors was diminished under a low glucose concentration in the *db/db* islets.

### The impact of treatment with metformin on the phosphorylation level of AMPK and the expression of the incretin receptors in islets *in vitro* and *in vivo*

We next assessed the effects of metformin on the expression of the incretin receptors in primary mouse islets *in vitro* and *in vivo*. Treatment with metformin significantly reduced the expression of the incretin receptors *in vitro* under a medium glucose concentration (11.1 mM) (Figure. S2A). To investigate the effect of metformin on the sensitivity of the islets to GLP-1 and GIP-stimulated insulin secretion, we examined insulin response to incretins at 2.8 and 11.1 mM glucose in isolated islets treated with vehicle or metformin at 11.1 mM glucose for 24 h. GLP-1 significantly increased GSIS in response to 11.1 mM glucose in the control islets, but not in the metformin-treated islets (Figure. S2B). We confirmed that the islet morphology and the insulin content in islets were unaffected by metformin treatment *in vitro* (Figure. S2C, D). The phosphorylation levels of AMPK in the control islets at 11.1 mM glucose were significantly decreased as compared with that at 2.8 mM glucose. As expected, treatment with metformin increased the phosphorylation levels of AMPK in the islets *in vitro* at 11.1 mM glucose (Figure. S2E).

We next evaluated the effect of administration of metformin on the expression of the incretin receptors in the islets *in vivo*. In contrast to the findings *in vitro*, treatment with metformin for 2 days significantly increased the expression of the incretin receptors in the islets *in vivo* (Figure. S3A). Pparα expression was also significantly increased by metformin treatment in the islets *in vivo* (Figure. S3A). We examined the effect of GLP-1 and GIP on GSIS in response to 2.8 or 11.1 mM glucose after treatment with metformin or vehicle control *in vivo* (Figure. S3B). GSIS in response to 11.1 mM glucose was significantly increased by GLP-1 and GIP in the islets of the vehicle- and metformin-treated mice, although the relative insulin response to incretins was comparable between the two groups (Figure. S3B). We next evaluated the phosphorylation levels of AMPK in the islets of the metformin-treated mice. Amazingly, the islets from the metformin-treated mice showed decreased phosphorylation level of AMPK (Figure. S3C). We also examined the phosphorylation levels of AMPK in the livers and skeletal muscles of the vehicle- and metformin-treated mice. Unlike in the islets, the phosphorylation levels of AMPK in the livers and skeletal muscles of the metformin-treated mice were significantly increased as compared to those in the untreated controls (Figure. S3C).

### Immunoblot analysis with the commercial antibody did not demonstrate the significant changes in the protein expression levels of incretin receptors

To assess the protein expression levels of GLP-1 receptor and GIP receptor in islets, we performed immunoblot analysis by the commercial available antibody. However, recent reports have shown that almost commercially available GLP-1 receptor antibodies do not detect authentic GLP-1 receptor protein, even using optimally enhanced methods [37], [38]. Our immunoblotting also showed that the anticipated bands of incretin receptors detected by the antibody were not affected by glucose concentration (Figure. S4), treatment with GKA (Figure. S5, S6), AMPK inhibitor (Figure. S7), AICAR (Figure. S8), db/db genotype (Figure. S9), or metformin (Figure. S2F, S3D).

## Discussion

In this study, we investigated the regulation of incretin receptor expressions in the pancreatic islets, and described that AMPK partly controlled them in a glucose concentration-dependent manner.

Although the expression of the incretin receptors in the islets was suppressed under chronic high concentration of glucose (22.2 mM), the glucose signal increased them at lower glucose concentrations (2.8–11.1 mM). A previous report also showed the expression of GLP-1 receptor in bell-shaped response to glucose [20]. This finding was supported by our results that GKA-induced glucose phosphorylation increased the expression of the expression of GLP-1 receptor in the islets under a low concentration of glucose (2.8 mM), while it decreased the expression under a medium concentration of glucose (11.1 mM). In addition, suppression of the incretin receptors expression under a high glucose concentration could be accounted for by the decreased expression of incretin receptors in the islets of *db/db* mice, a model of hyperglycemia, as shown in a previous report [8]. By what mechanism did glucose signal increase the expression of the incretin receptors under lower glucose concentrations and suppress the expression under a high glucose concentration? A previous report showed that GIP receptor expression was glucose-dependently increased in isolated rat islets incubated for 48 hours, and the result suggested that the rapid increase in GIP receptor could contribute to the maintenance of insulin output during the elevated metabolic demand induced by glucose elevation [8]. In contrast, decrease in the expression of the incretin receptors after exposure to high glucose concentrations has also been reported from previous studies [8], [20], [39]. Xu *et al* indicated that PKCα may be involved in the downregulation of the GLP-1 receptor expression at 20 mM glucose [8]. Cheong *et al.* showed that a high glucose concentration (30 mM glucose) reduced the GLP-1 receptor expression through endoplasmic reticulum (ER) stress [39]. Rajan *et al.* recently showed that downregulation of GLP-1 receptor signaling at a high glucose concentration was due to the reduction of cell surface trafficking of GLP-1 receptor by small ubiquitin-related modifier protein (SUMO) [40]. Persistence of hyperglycemia may interfere with incretin receptor signaling by an additive effect of the transcriptional attenuation and the posttranslational modification of receptors. The change in expression of GIP receptor was somewhat different as compared with those found with the GLP-1 receptor expression by treatment with GKA at 11.1 mM glucose. Xu *et al* showed that increases in GIP receptor expression were found in the short-term glucose infusion model and cultured islets with exposure to high glucose, although reduction in GLP-1 receptor was observed in the models [8]. These differences in the expression of GIP and GLP-1 receptor may be due to timing and we need to elucidate the differences in future.

The finding in our study, demonstrating that the phosphorylation of AMPK was decreased by increasing glucose concentrations, although the expression of the incretin receptors was in bell-shaped response to glucose, suggested that the glucose signal induced-regulation of incretin receptors expression appeared to be an AMPK-independent pathway. However, under a high glucose concentration, above-mentioned factors largely seem to affect the regulation of the expression of the incretin receptors. Therefore, our hypothesis was that AMPK was partly involved in the regulation of the incretin receptors expression under lower glucose concentrations (2.8–11.1 mM). By contrast, we also demonstrated that the enhancing effect of AMPK inhibitor on the expression of the incretin receptors was diminished in *db/db* islets under chronic hyperglycemic conditions. The data in *db/db* islets also suggested that AMPK is not involved in the regulation of the incretin receptors expression under a high glucose concentration. It has been shown that not only hyperglycemia, but also ER stress [41] and oxidative stress [42], [43] may contribute to β cell dysfunction in the *db/db* mice. Therefore, it is possible that AMPK activity fails to contribute to the regulation of the expression of the incretin receptors under ER or oxidative stress conditions. Since the disruption of leptin receptor expression in the pancreas directly affects β cell function [44], the impact of the leptin receptor-mediated mechanisms on insulin secretion in islets may possibly reflect the diminished effects of AMPK regulation on the incretin receptors expression. Collectively, these results suggested that AMPK was involved in the regulation in the expression of incretin receptors under low and medium glucose concentrations, but not under a high glucose concentration.

The fact that AICAR failed to significantly decrease the expression of the incretin receptors, suggests that the regulation in the expression appear to be an AMPK-independent mechanism. In this study, we treated isolated islets with AICAR under a medium glucose concentration (11.1 mM) to suppress the endogenous enzyme to some extent. However, phosphorylation of AMPK in islet was not fully suppressed under a medium glucose concentration (Figure 1A). Therefore, it may be difficult to assess the direct effect of AICAR on the expression of the incretin receptors in islet under the condition. We need to assess the impact of AICAR on the expression of the incretin receptors in islets under other conditions to suppress the phosphorylation of AMPK to a large extent.

The results in our study also demonstrated that inhibition of AMPK increased insulin secretion, as shown in a previous report [28]. The previous finding showed that inhibition of AMPK with DN-AMPK led to the activation of insulin secretion via Ca^2+^-independent mechanism [28]. It is possible that AMPK dephosphorylation-induced insulin secretion may make it difficult to evaluate the insulin response to incretins in islets treated with AMPK inhibitor in our current models. Moreover, intact islets contain not only β cells but α or δ cells. Although, a previous report showed that the expression of GLP-1 receptor in alpha cells was <0.2% of that in β cells [45], AMPK is regulated by glucose in pancreatic alpha cells, and increases in AMPK activity are sufficient and necessary for the stimulation of glucagon release [46]. Therefore, we need to assess the effect of AMPK regulation in alpha cells on the incretin receptors expression in islets in the future.

The correlation between the expression of the incretin receptors and the phosphorylation level of AMPK *in vitro* and *in vivo* suggests that metformin may partly regulate the expression in islets via AMPK-dependent pathway. However, the inhibitory effect of metformin on mitochondrial electron transport chain also led to AMPK activation secondarily [47], therefore the secondary effect of metformin on AMPK activation may partially regulate the incretin receptors expression in islets in this study. Moreover, the time for isolating islets *in vivo* and the differences in the concentrations of metformin between *in vitro* and *in vivo* may affect the discrepant results. By contrast, the finding that metformin and AICAR increased AMPK phosphorylation, but only metformin significantly decreased the expression of the incretin receptors, suggests that an AMPK-independent mechanism may be involved in the regulation of the expression by metformin. We should further assess what mechanism underlying the regulation of the expression of the incretin receptors by metformin using other experimental models.

In this study, we showed that there were no significant changes in the protein expression of the incretin receptors in islets between control group and experimental group in each experiment. There is a possibility that the commercially available antibody of GLP-1 receptor failed to detect authentic GLP-1 receptor protein. It is well-known that the GLP-1 receptor and other GPCRs are membrane-spanning molecules. However, it has been shown that pancreatic islets are known to frequently give rise to false-positive staining in immunohistochemistry studies [48]. More recently, Panjwani *et al.* have shown that the commercial antibodies could not detect the native GLP-1R protein in conventional Western blot analysis, and the antibodies did detect several immunoreactive bands ranging in size from approximately 54–65 kDa even in lung extracts from both Glp1r^+/+^ and Glp1r^−/−^ mice [37]. Pyke *et al.* have also shown that the commercial antibodies recommended for IHC reacted with equal intensity with both GLP-1R-transfected and untransfected cells and, when further diluted to a concentration where there was negligible staining of untransfected cells, did not react with GLP-1R-transfected cells [38]. Based upon the fact, it is possible that the commercial antibody used in this study detected non-specific bands, but not authentic GLP-1 receptor protein. Therefore, we would very much like to assess the reliable GLP-1 receptor protein expression data using other technical challenge. In previous studies, many groups reported that a lack of specificity of commercial antibodies against GPCRs (G protein-coupled protein receptors) was shown to be the rule rather than the exception [49]–[54]. The lack of specificity of GPCRs antibodies may also apply to the GIP receptor. However, we also should assess the protein expression of GIP receptor as with that of GLP-1 receptor using other technical procedure.

In summary, we show here that AMPK is involved in the regulation in the expression of incretin receptors under a low glucose concentration. The results of the current study have important implications in relation to the design of new therapies targeting the expression of the incretin receptors, however, further research is needed to clarify the underlying mechanisms.

## Supporting Information

Figure S1 **The effect of adenoviral infection on the apoptosis in islets.** Trypsinized islet cells of indicated groups were subjected to a TUNEL assay. Insulin is stained green and TUNEL-positive nuclei are stained red. DAPI (blue) and differential interference contrast (DIC) images were also merged. TUNEL staining was performed using the ApopTag In Situ Detection Kit (Chemicon). For TUNEL staining, at least 40 islets per trypsinized islets group attached to poly-L-lysine coated coverslips (Falcon) were analyzed using the FLUOVIEW FV 1000-D confocal laser scanning microscope (OLYMPUS) to assess the proportion of immunostained nuclei among the insulin-positive cells.
(TIF)

Figure S2 **The impact of treatment with metformin on the phosphorylation level of AMPK and the expressions of the incretin receptors in islets** ***in vitro*** **.** A), B) Isolated islets were incubated with or without 1 mM metformin (Dainippon Sumitomo Pharma, Osaka, Japan) for 24 h in the presence of 2.8 or 11.1 mM glucose. A) The Glp1, Gip receptor and Pparα expressions in the islets were determined by real-time quantitative RT-PCR and normalized to the expression level of β actin mRNA and to the vehicle-treated control samples at 2.8 mM glucose (n = 7–8). *P<0.05. B) Insulin secretion by the islets treated with metformin or vehicle (ctl) in the presence of 11.1 mM glucose for 24 h with or without addition of 10 nM GLP-1 or 10 nM GIP. The results are expressed in ng of insulin/10 islets/90 min (n = 4). * P<0.05 and ** P<0.01. C), D) Isolated islets were incubated with or without 1 mM metformin for 24 h in the presence of 11.1 mM glucose. C) Islet morphology of the vehicle- and metformin-treated islets. D) The insulin content in the islets was determined after acid ethanol extraction (n = 3). White bars, vehicle; Black bars; metformin. E) Total islet extracts from the isolated islets were subjected to immunoblotting for p-AMPKα (Thr^172^) and AMPKα. The intensities of the signals were quantified by densitometry and normalized to the vehicle-treated control samples at 2.8 mM glucose (n = 3). The results shown are the means of three independent experiments. F) Total cell extracts from the isolated islets were subjected to immunoblotting for p-AMPKα (Thr^172^), AMPKα, anti-GLP1R antibody, and β actin.
(TIF)

Figure S3 **The impact of treatment with metformin on the phosphorylation level of AMPK and the expressions of the incretin receptors in islets** ***in vivo*** **.** Mice were given metformin in drinking water at 300 mg/kg daily or standard drinking water for 48 hours. Four hours prior to the islet isolation, the food was removed from the cages and the mice were orally administrated metformin (75 mg/kg) or vehicle (water) [14]. A) The Glp1, Gip receptor and Pparα expressions in the islets were determined by real-time quantitative RT-PCR and normalized to the expression level of β actin mRNA and to the samples in vehicle-treated mice (n = 6–7). Experiments were performed on mice treated with metformin or vehicle (ctl). *P<0.05 and ** P<0.01. B) Insulin secretion from the islets of mice treated with metformin or vehicle (ctl) in the presence of 2.8 mM or 11.1 mM glucose, with or without addition of 10 nM GLP-1 or 10 nM GIP. The results are expressed as ng of insulin/10 islets/90 min (n = 4). * P<0.05 and ** P<0.01. C) Freshly isolated total islets, the extracted liver and skeletal muscle tissues from the vehicle or metformin-treated mice were subjected to immunoblotting for p-AMPKα (Thr^172^) and AMPKα. The intensities of the signals were quantified by densitometry and normalized to the samples in vehicle-treated mice (n = 3). The results shown are the means of three independent experiments. D) Total cell extracts from the isolated islets were subjected to immunoblotting for p-AMPKα (Thr^172^), AMPKα, anti-GLP1R antibody, and β actin (n = 3).
(TIF)

Figure S4 **The effect of glucose signal on the protein expression of the incretin receptors in islets.** Isolated islets were incubated for 24 h in the presence of 2.8, 5.6, 11.1 or 22.2 mM glucose. Total cell extracts from the isolated islets were subjected to immunoblotting for p-AMPKα (Thr^172^), AMPKα, anti-GLP1R antibody, anti-GIPR antibody, and GAPDH.
(TIF)

Figure S5 **The effect of glucokinase activator on the protein expression of the incretin receptors under a low glucose concentration.** Isolated islets were incubated with DMSO (ctl) or GKA (G: 30 µM compound A) in the presence of 2.8 mM glucose for 24 hours. Total cell extracts from the isolated islets were subjected to immunoblotting for p-AMPKα (Thr^172^), AMPKα, anti-GLP1R antibody, anti-GIPR antibody, and GAPDH (n = 3).
(TIF)

Figure S6 **The effect of glucokinase activator on the protein expression of the incretin receptors under a medium glucose concentration.** Isolated islets were incubated with DMSO (ctl) or GKA (G: 30 µM compound A) in the presence of 11.1 mM glucose for 24 hours. Total cell extracts from the isolated islets were subjected to immunoblotting for p-AMPKα (Thr^172^), AMPKα, anti-GLP1R antibody, anti-GIPR antibody, and GAPDH (n = 3).
(TIF)

Figure S7 **The effect of pharmacologic modulation of AMPK phosphorylation on the protein expressions of the incretin receptors under a low glucose concentration.** Isolated islets were treated for 24 h with vehicle (Veh; DMSO) or the AMPK inhibitor (AMPKi; 40 µM compound C) in the presence of 2.8 mM glucose. Total cell extracts from the isolated islets were subjected to immunoblotting for p-AMPKα (Thr^172^), AMPKα, anti-GLP1R antibody, anti-GIPR antibody, and GAPDH (n = 3).
(TIF)

Figure S8 **The effect of pharmacologic modulation of AMPK phosphorylation on the protein expression of the incretin receptors under a medium glucose concentration.** Isolated islets were treated for 24 h with vehicle (V, water) or AICAR (A, 1 mM) in the presence of 11.1 mM glucose. Total cell extracts from the isolated islets were subjected to immunoblotting for p-AMPKα (Thr^172^), AMPKα, anti-GLP1R antibody, anti-GIPR antibody, and GAPDH (n = 4)
(TIF)

Figure S9 **The impact of pharmacologic modulation of AMPK phosphorylation on the protein expressions of the incretin receptors in** ***db/db*** **mice.** Isolated islets from 7-week-old *db/+* and *db/db* mice were pre-treated for 15 min with vehicle (Veh; DMSO) or an AMPK inhibitor (AMPKi; 40 µM compound C) in the presence of 2.8 mM glucose, or with vehicle (Veh; DMSO) in the presence of 11.1 mM glucose, followed by the addition of vehicle (Veh; water) for an additional 24 h. Total cell extracts from the isolated islets were subjected to immunoblotting for p-AMPKα (Thr^172^), AMPKα, anti-GLP1R antibody, anti-GIPR antibody, and GAPDH.
(TIF)
