# Supplementary figures and images for: AMPK Is Involved in the Regulation of Incretin Receptors Expression in Pancreatic Islets under a Low Glucose Concentration
Source: PLoS One. 2013 May 22;8(5):e64633. doi: 10.1371/journal.pone.0064633 (PMC3661597; doi:10.1371/journal.pone.0064633)

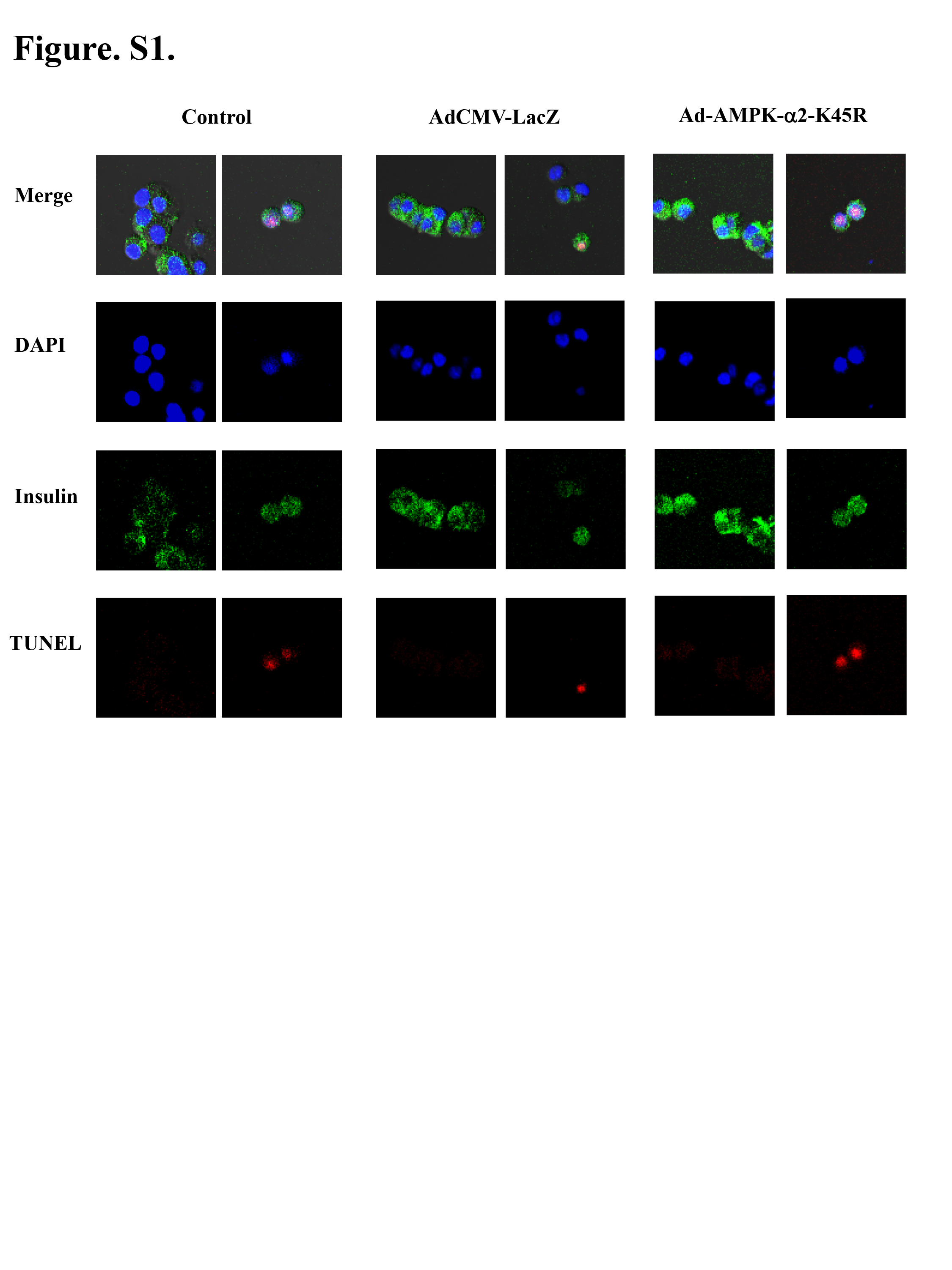

Supplement: Figure S1 — The effect of adenoviral infection on the apoptosis in islets. Trypsinized islet cells of indicated groups were subjected to a TUNEL assay. Insulin is stained green and TUNEL-positive nuclei are stained red. DAPI (blue) and differential interference contrast (DIC) images were also merged. TUNEL staining was performed using the ApopTag In Situ Detection Kit (Chemicon). For TUNEL staining, at least 40 islets per trypsinized islets group attached to poly-L-lysine coated coverslips (Falcon) were analyzed using the FLUOVIEW FV 1000-D confocal laser scanning microscope (OLYMPUS) to assess the proportion of immunostained nuclei among the insulin-positive cells. (TIF) [file pone.0064633.s001.tif]

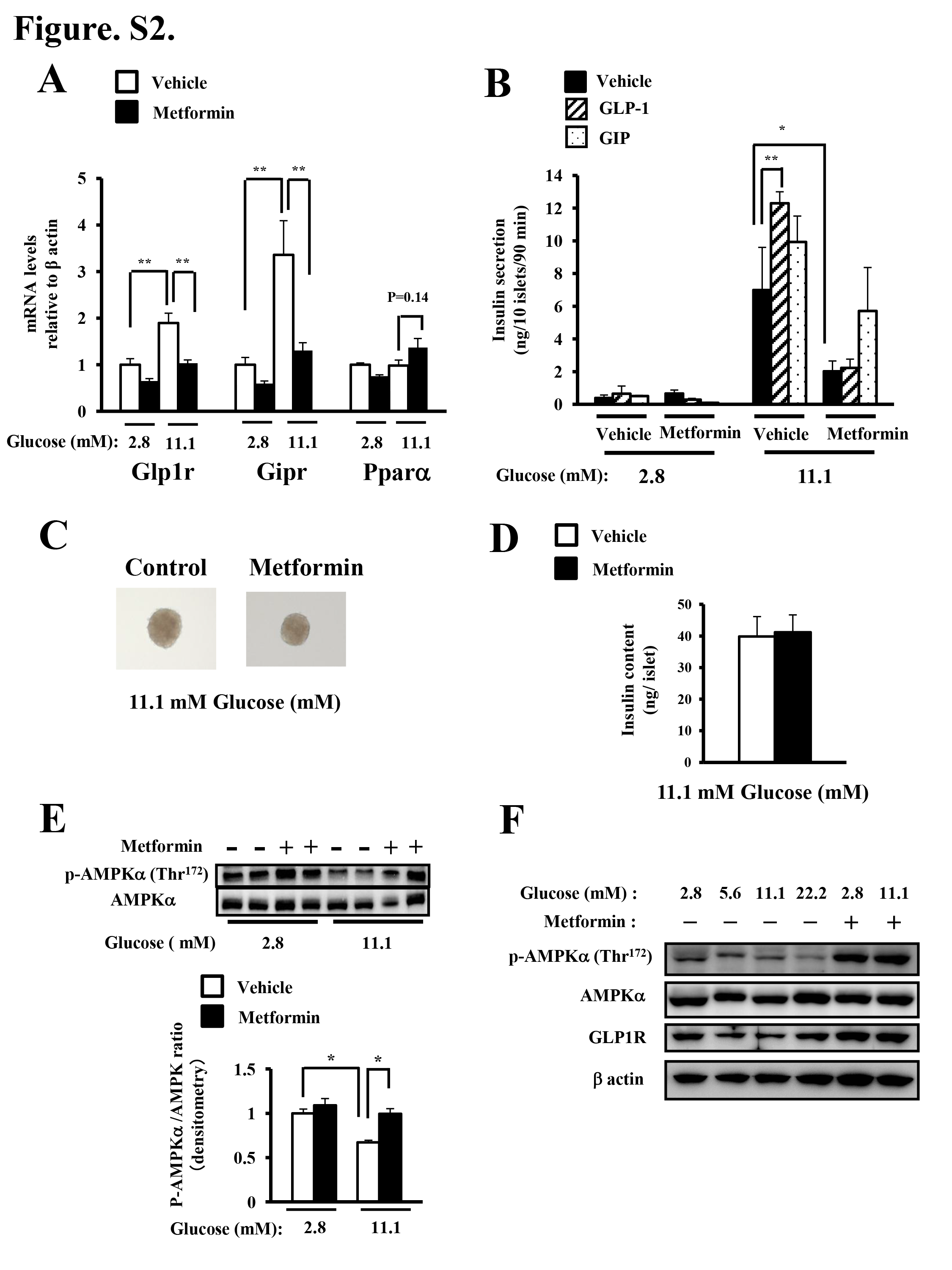

Supplement: Figure S2 — The impact of treatment with metformin on the phosphorylation level of AMPK and the expressions of the incretin receptors in islets in vitro . A), B) Isolated islets were incubated with or without 1 mM metformin (Dainippon Sumitomo Pharma, Osaka, Japan) for 24 h in the presence of 2.8 or 11.1 mM glucose. A) The Glp1, Gip receptor and Pparα expressions in the islets were determined by real-time quantitative RT-PCR and normalized to the expression level of β actin mRNA and to the vehicle-treated control samples at 2.8 mM glucose (n = 7–8). *P<0.05. B) Insulin secretion by the islets treated with metformin or vehicle (ctl) in the presence of 11.1 mM glucose for 24 h with or without addition of 10 nM GLP-1 or 10 nM GIP. The results are expressed in ng of insulin/10 islets/90 min (n = 4). * P<0.05 and ** P<0.01. C), D) Isolated islets were incubated with or without 1 mM metformin for 24 h in the presence of 11.1 mM glucose. C) Islet morphology of the vehicle- and metformin-treated islets. D) The insulin content in the islets was determined after acid ethanol extraction (n = 3). White bars, vehicle; Black bars; metformin. E) Total islet extracts from the isolated islets were subjected to immunoblotting for p-AMPKα (Thr172) and AMPKα. The intensities of the signals were quantified by densitometry and normalized to the vehicle-treated control samples at 2.8 mM glucose (n = 3). The results shown are the means of three independent experiments. F) Total cell extracts from the isolated islets were subjected to immunoblotting for p-AMPKα (Thr172), AMPKα, anti-GLP1R antibody, and β actin. (TIF) [file pone.0064633.s002.tif]

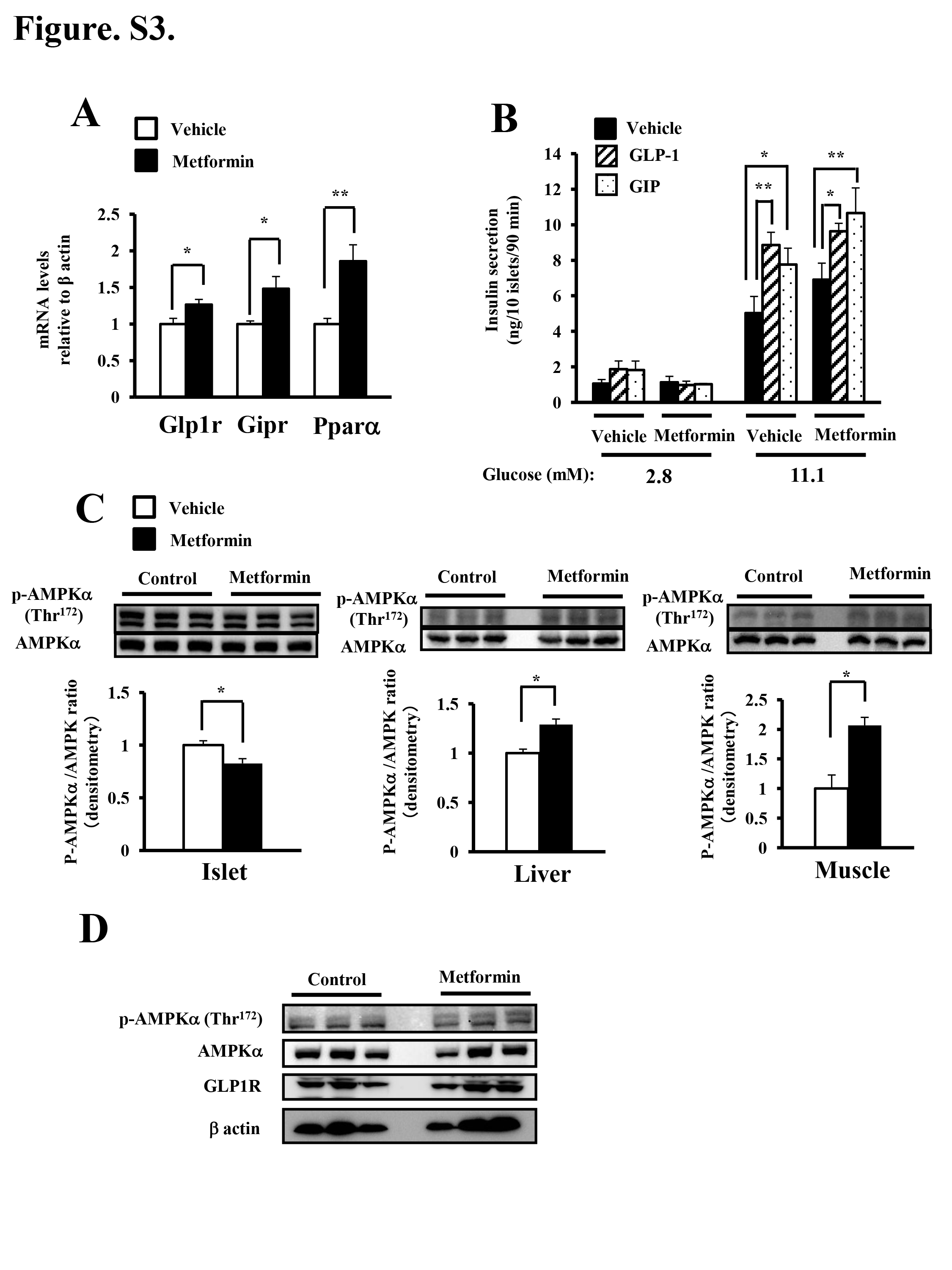

Supplement: Figure S3 — The impact of treatment with metformin on the phosphorylation level of AMPK and the expressions of the incretin receptors in islets in vivo . Mice were given metformin in drinking water at 300 mg/kg daily or standard drinking water for 48 hours. Four hours prior to the islet isolation, the food was removed from the cages and the mice were orally administrated metformin (75 mg/kg) or vehicle (water) [14]. A) The Glp1, Gip receptor and Pparα expressions in the islets were determined by real-time quantitative RT-PCR and normalized to the expression level of β actin mRNA and to the samples in vehicle-treated mice (n = 6–7). Experiments were performed on mice treated with metformin or vehicle (ctl). *P<0.05 and ** P<0.01. B) Insulin secretion from the islets of mice treated with metformin or vehicle (ctl) in the presence of 2.8 mM or 11.1 mM glucose, with or without addition of 10 nM GLP-1 or 10 nM GIP. The results are expressed as ng of insulin/10 islets/90 min (n = 4). * P<0.05 and ** P<0.01. C) Freshly isolated total islets, the extracted liver and skeletal muscle tissues from the vehicle or metformin-treated mice were subjected to immunoblotting for p-AMPKα (Thr172) and AMPKα. The intensities of the signals were quantified by densitometry and normalized to the samples in vehicle-treated mice (n = 3). The results shown are the means of three independent experiments. D) Total cell extracts from the isolated islets were subjected to immunoblotting for p-AMPKα (Thr172), AMPKα, anti-GLP1R antibody, and β actin (n = 3). (TIF) [file pone.0064633.s003.tif]

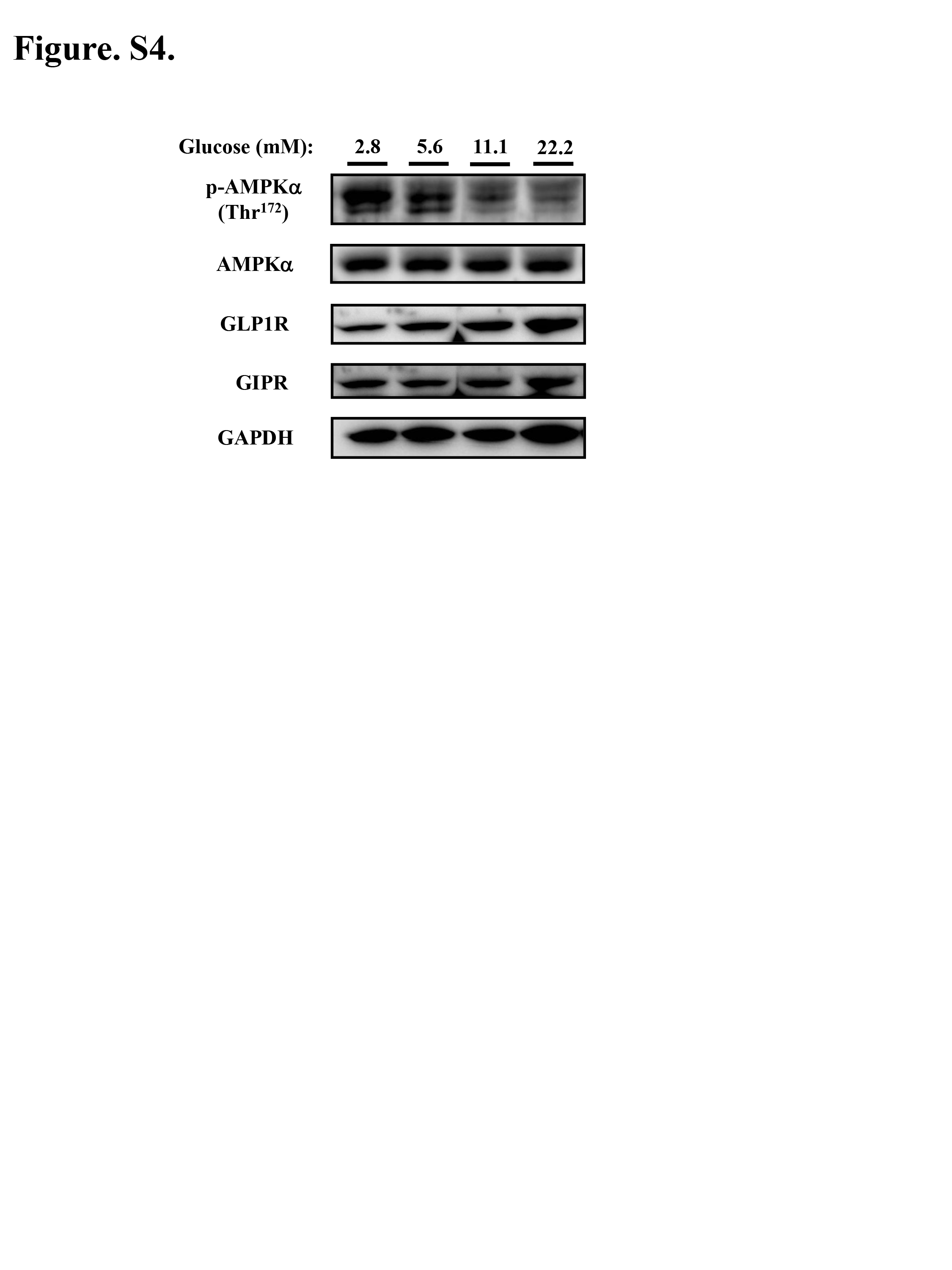

Supplement: Figure S4 — The effect of glucose signal on the protein expression of the incretin receptors in islets. Isolated islets were incubated for 24 h in the presence of 2.8, 5.6, 11.1 or 22.2 mM glucose. Total cell extracts from the isolated islets were subjected to immunoblotting for p-AMPKα (Thr172), AMPKα, anti-GLP1R antibody, anti-GIPR antibody, and GAPDH. (TIF) [file pone.0064633.s004.tif]

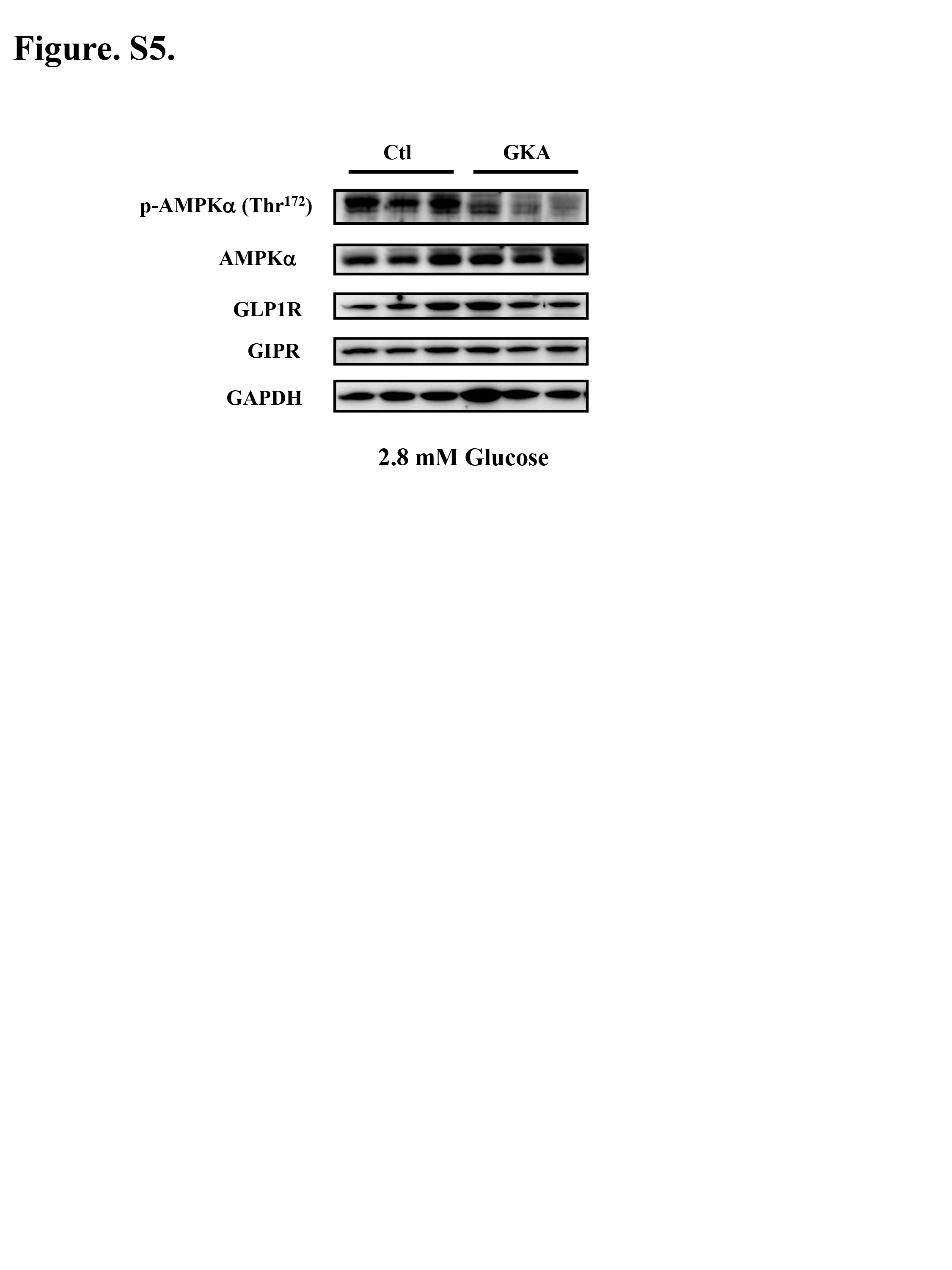

Supplement: Figure S5 — The effect of glucokinase activator on the protein expression of the incretin receptors under a low glucose concentration. Isolated islets were incubated with DMSO (ctl) or GKA (G: 30 µM compound A) in the presence of 2.8 mM glucose for 24 hours. Total cell extracts from the isolated islets were subjected to immunoblotting for p-AMPKα (Thr172), AMPKα, anti-GLP1R antibody, anti-GIPR antibody, and GAPDH (n = 3). (TIF) [file pone.0064633.s005.tif]

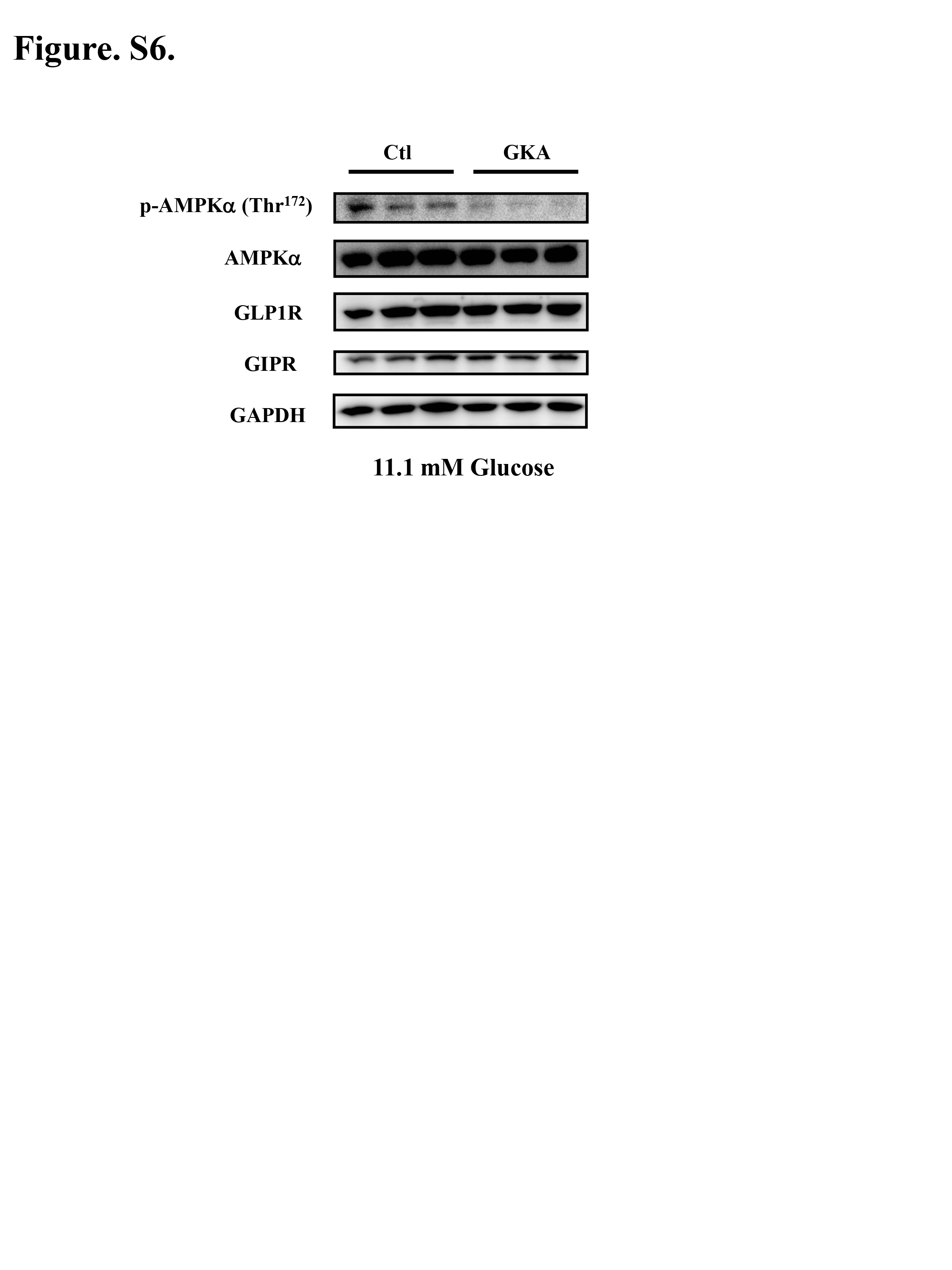

Supplement: Figure S6 — The effect of glucokinase activator on the protein expression of the incretin receptors under a medium glucose concentration. Isolated islets were incubated with DMSO (ctl) or GKA (G: 30 µM compound A) in the presence of 11.1 mM glucose for 24 hours. Total cell extracts from the isolated islets were subjected to immunoblotting for p-AMPKα (Thr172), AMPKα, anti-GLP1R antibody, anti-GIPR antibody, and GAPDH (n = 3). (TIF) [file pone.0064633.s006.tif]

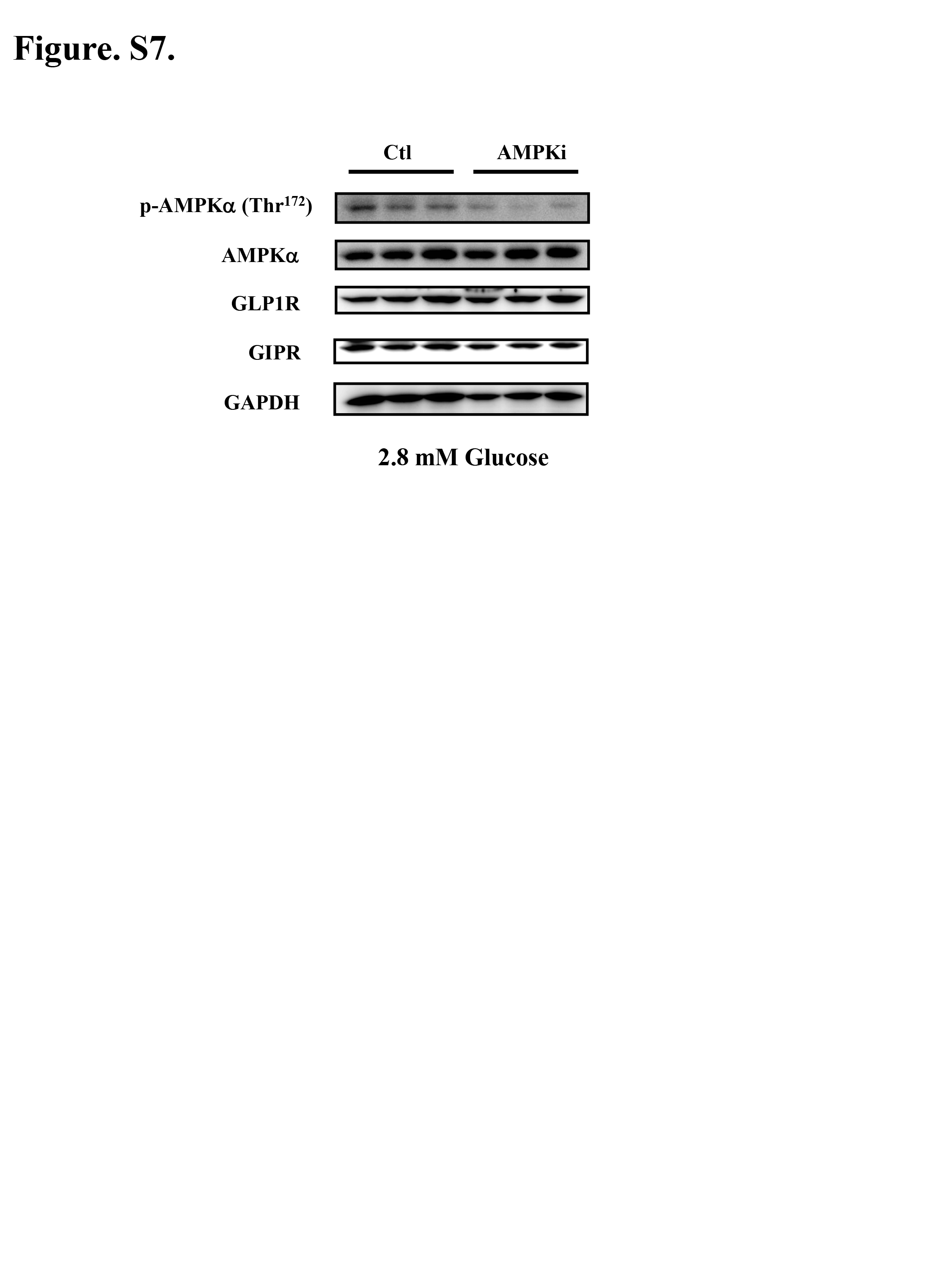

Supplement: Figure S7 — The effect of pharmacologic modulation of AMPK phosphorylation on the protein expressions of the incretin receptors under a low glucose concentration. Isolated islets were treated for 24 h with vehicle (Veh; DMSO) or the AMPK inhibitor (AMPKi; 40 µM compound C) in the presence of 2.8 mM glucose. Total cell extracts from the isolated islets were subjected to immunoblotting for p-AMPKα (Thr172), AMPKα, anti-GLP1R antibody, anti-GIPR antibody, and GAPDH (n = 3). (TIF) [file pone.0064633.s007.tif]

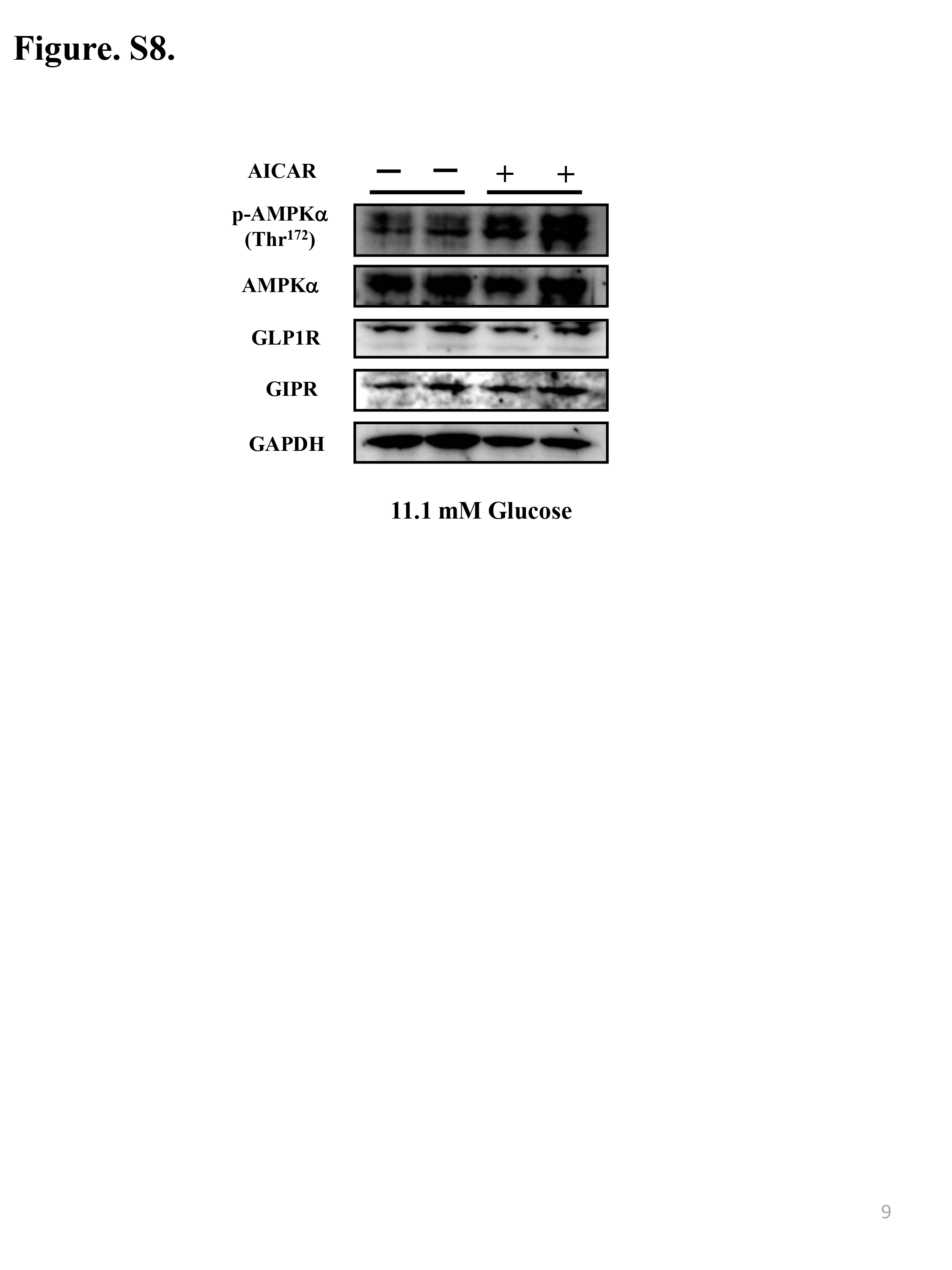

Supplement: Figure S8 — The effect of pharmacologic modulation of AMPK phosphorylation on the protein expression of the incretin receptors under a medium glucose concentration. Isolated islets were treated for 24 h with vehicle (V, water) or AICAR (A, 1 mM) in the presence of 11.1 mM glucose. Total cell extracts from the isolated islets were subjected to immunoblotting for p-AMPKα (Thr172), AMPKα, anti-GLP1R antibody, anti-GIPR antibody, and GAPDH (n = 4) (TIF) [file pone.0064633.s008.tif]

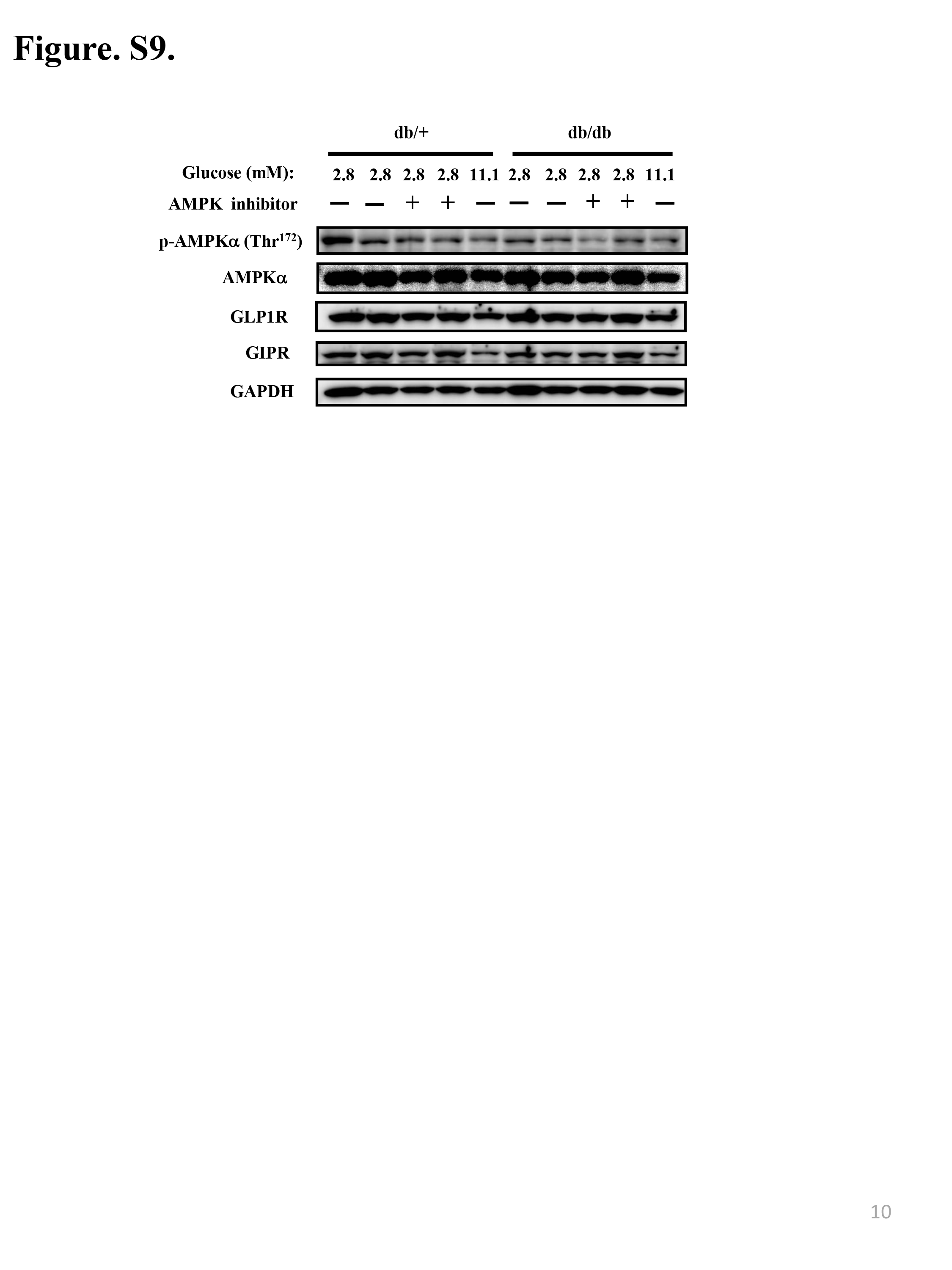

Supplement: Figure S9 — The impact of pharmacologic modulation of AMPK phosphorylation on the protein expressions of the incretin receptors in db/db mice. Isolated islets from 7-week-old db/+ and db/db mice were pre-treated for 15 min with vehicle (Veh; DMSO) or an AMPK inhibitor (AMPKi; 40 µM compound C) in the presence of 2.8 mM glucose, or with vehicle (Veh; DMSO) in the presence of 11.1 mM glucose, followed by the addition of vehicle (Veh; water) for an additional 24 h. Total cell extracts from the isolated islets were subjected to immunoblotting for p-AMPKα (Thr172), AMPKα, anti-GLP1R antibody, anti-GIPR antibody, and GAPDH. (TIF) [file pone.0064633.s009.tif]
